# Advances in the applications of monoclonal antibodies in clinical oncology. London, 6-8 May 1987. Abstracts.

**DOI:** 10.1038/bjc.1987.235

**Published:** 1987-10

**Authors:** 


Br. J. Cancer (1987), 56, 509-530 © The Macmillan Press Ltd., 1987

### Advances in the applications of monoclonal antibodies in clinical oncology

University of London Royal Postgraduate Medical School

*Held at the Wolfson Institute, 6–8 May, 1987*

### Abstracts of oral presentations

### The potential of hybrid antibodies secreted by hybrid-hybridomas in tumour therapy

M. Clark, L. Gilliland & H. Waldmann

*Division of Immunology, Department of Pathology, University of Cambridge, Cambridge CB2 1QP, UK.*

The cell fusion of two Ig producing cell lines may result in the codominant expression of all of the parental cell derived Ig chains. For a cell making two Ig heavy chains and two Ig light chains a random association of all chains results in 10 possible configurations of secreted antibody species. In practice, not all the combinations of chains are observed at the predicted frequencies for a random association but the mixture is still usually complex. The interest in these antibodies for therapy stems from the biological properties of different species within the complex mixture. In addition to the properties which depend upon the interaction of different Ig isotypes with human effector mechanisms, there are unique properties which result from the different binding specificities for antigen. For example, certain species of antibody are monovalent for binding to a given antigen. Such monovalent antibodies with specificities for modulating cell surface antigens seem to be more efficient than the equivalent bivalent antibodies at lysing target cells with complement. Another of the components of the mixture has dual specificity for the two different antigens recognised by the parental antibody types. These bi-specific antibodies are capable of targetting drugs or toxins to cells expressing appropriate cell surface antigens. Also of interest is the ability of these bi-specific antibodies to induce very potent tumour cell killing by activated T-cell blasts when the bi-specific antibody is used to crosslink a component of the T-cell receptor complex and a suitable cell surface antigen on the target cell.

### Leucocyte differentiation antigens

A. McMichael

*Nuffield Department of Medicine, John Radcliffe Hospital, Headington, Oxford OX3 9DU, UK.*

The results of the Third International Workshop and Conference on Human Leucocyte Differentiation Antigens will be presented. There are now a total of 50 named leucocyte differentiation antigens. They have been defined by exchange of monoclonal antibodies followed by comparisons of binding, tissue staining, immunoprecipitation and functional studies. For some of the antigens a function has been defined, others have no known function as yet. There are problems in defining lineages on normal and abnormal cells.

### Recombinant antibodies

L. Riechman & G. Winter

*Medical Research Council Laboratory of Molecular Biology, Cambridge CB2 2QH, UK.*

Immunotherapy has been used to treat pathogens and to provide passive immunity. However, there can be problems, i.e., the 'neutralising' antibody is only a small fraction of the total immunoglobulin, human antisera may be infected with viruses such as AIDS and hepatitis, and antibodies from animal sources are recognised as foreign. These problems should not occur if human monoclonal antibodies are used. Unfortunately, the production of human monoclonal antibodies has proved difficult. Therefore, we have attempted, via recombinant DNA technology to turn mouse or rat monoclonal antibodies into 'human' antibodies.

At its simplest, variable regions from mouse or rat antibodies conferring the antigen specificity can be combined with human constant domains, resulting in a chimeric antibody having predetermined specificity and effector function (Neuberger *et al. Nature,* **314**: 268, 1985). Although such antibodies may yet prove to be therapeutically useful, an immune response of the recipient to mouse or rat variable regions seems probable. Therefore the parts of the animal variable domain conferring the antigen specificity have been transplanted into the framework regions of a human variable domain. We have applied this technique to construct 'humanised' antibodies binding a small hapten (Jones *et al. Nature,* **321:** 522, 1986) to lysozyme (unpublished) and to a cell surface antigen, CAMPATH 1 (unpublished).

### Coordinate elevation of serum markers in ovarian cancer but not in benign disease

R.C. Bast, Jr., S. Knauf, A.A. Epenetos, M. Tanner, T. Klug, J. Soper, W. Creasman, S. Gall, R.C. Knapp, V. Zurawski, Jr., J. Schlom, D. Kufe & R.E. Ritts

*Duke University Medical Center, Durham, NC; University of Rochester, Rochester, NY, USA; Hammersmith Hospital, London, UK; Centocor, Malvern, PA; National Cancer Institute, Bethesda, MD; Dana Farber Cancer Institute, Boston, MA; Mayo Clinic, Rochester, MN, USA.*

Effective screening for occult ovarian cancer requires tests that are both sensitive and specific. Data so far suggests that CA-125 is elevated in most patients with ovarian cancer. As a single test, however, CA-125 is not sufficiently specific to

Course organiser: A.A. Epenetos, Royal Postgraduate Medical School and Hammersmith Hospital, London W12 0HS.

permit cost effective screening of an apparently healthy population. In order to develop a more specific screening, multiple serum markers have been assayed with a panel of sera from 51 ovarian cancer patients and from 50 individuals with benign disease whose serum CA-125 levels exceeded $35\,U\,ml^{-1}$. Among the patients with ovarian cancer, elevations of CA-125 ($>65\,U\,ml^{-1}$) were observed in 63%, CA15-3 ($>30\,U\,ml^{-1}$) in 59%, TAG 72.3 ($>10\,U\,ml^{-1}$) in 47%, placental alkaline phosphatase (PLAP) in 31%, HMFG1 in 76%, HMFG2 in 63% and NB/70K in 57%. All patients with elevated CA-15-3 or TAG 72.3 also had a CA-125 $>35\,U\,ml^{-1}$. Among the 50 'false positive' sera selected from 4,947 donors, CA-125 was >35% in 100%, NB/70K was elevated in 62%, TAG 72.3 in 6% and CA-15-3 in 2%. PLAP also appeared quite promising in that elevated enzyme levels were not found in the 'false positive' group. HMFG1 was elevated in 26% and HMFG2 in 12%. Among ovarian cancer patients with CA-125 $>65\,U\,ml^{-1}$, TAG 72.3 or CA-15-3 was elevated in 83%. In the 'false positive' group, only 5% of patients had elevations of one or other marker. Prospective studies are required to test whether serum markers in combination will prove sufficiently specific to justify their use in individuals at risk for ovarian cancer.

### Radiolabelled monoclonal anti-CEA antibodies and fragments in colon carcinoma – diagnostic and therapeutic approach

J.P. Mach, J. Pettavel, A. Bishop-Delaloye & B. Delaloye

*Institute of Biochemistry, University of Lausanne, CA 10066 Epalinge and Div. of Nuclear Medicine, CH-1011 Lausanne, Switzerland.*

Thirty-one patients with known colo-rectal carcinoma were injected with I-123 labelled $F(ab')_2$ or Fab ($n=17$) fragments from monoclonal antibodies (MAb) anti-CEA. Patients were examined by ECT at 6, 24 and sometimes 48 h after injection using a rotating dual head scintillation camera. As recently reported (Delaloye *et al., J. Clin. Invest,* **77,** 301, 1986), all 23 primary tumours and local recurrences except one were clearly visualised on at least two sections of different tomographic planes. Interestingly, 9 of these patients had almost normal circulating CEA levels and 3 of the visualised tumours weigh only 3 to 5 g. Following encouraging preliminary results of tumour therapy in nude mice, showing that anti-CEA MAbs labelled with therapeutic doses of I-131 could inhibit the growth of human colon carcinoma xenografts or provoke tumour regression. Seven patients with liver metastases of colon carcinoma were injected into the hepatic artery with MAbs anti-CEA labelled with 100 mCi I-131. We observed no side effects and good localisation of I-131 MAbs in liver metastases as documented by ECT, but we have not yet obtained definite evidence of tumour regression.

### The use of immunocytochemistry in surgical pathology

G.W.H. Stamp

*Department of Histopathology, Hammersmith Hospital, London W12 0HS, UK.*

Immunocytochemistry has now established itself as a valuable procedure in surgical pathology. A variety of antibodies, both polyclonal and monoclonal are in use, and they are usually well characterised and commercially available. The illustrated examples are arbitrarily grouped into lymphoreticular markers, cell membrane antigens such as HMFG1 and 2, cytoskeletal components (intermediate filaments and actin), enzymes (NSE, prostatic acid phosphatase, PLAP), cytoplasmic protein (e.g. S-100), extracellular base membrane components (collagen type 4 and laminin), 'appropriate' cell products (peptide hormones, thyroglobulin, myoglobin, factor VIII-related antigen) and 'inappropriate' products including 'tumour markers' (CEA, AFP, HCG hormones).

Problems arising from inadequately prepared material and technical aspects leading to possible misleading results are considered, and the necessity for close liason between the referring clinician and surgical pathologist is emphasised.

### Monoclonal antibodies at EM level

J. Polak

*Department of Histochemistry, Royal Postgraduate Medical School, Hammersmith Hospital, London W12 0HS, UK.*

The ability to monitor cellular events and to identify, morphologically, sub-cellular components involved in particular processes has greatly advanced since the advent of monoclonal antibodies and their use in electron immunocytochemistry. For instance, using simultaneous monoclonal and polyclonal antibodies (to proteins and enzymes capable of monitoring intracellular pH) on specially prepared tissues it is now possible to visualise intracellular pathways involved in protein synthesis.

The demonstration of co-existence of several substances (e.g., active peptides) in a single cell has been greatly aided by the use of monoclonal and polyclonal antibodies in combination. For light microscopical studies, different reaction products are revealed by the use of differently coloured chromagens. For electron microscopy, monoclonal and polyclonal antibodies are labelled with gold particles of different sizes and are used on ultra-thin sections (the on-grid immunolabelling or post-embedding method).

Low numbers of binding sites on cellular membranes can now be visualised at the electron microscopical level, by the use of specific monoclonal antibodies to receptors or by the construction of divalent forms of antigens that can react with the receptor and subsequently to a monoclonal antibody. Again, the reaction is revealed by gold labelling procedures.

### Results of 1st international workshop on small cell lung cancer antigens

P.C.L. Beverley, L.G. Borbrow & R.L. Souhami

*ICRF Human Tumour Immunology Group, University College Hospital, Radiotherapy Department, University College Hospital, London WC1E, UK.*

The workshop used methods, developed in three international workshops on leucocyte differentiation antigens, to group together clusters of monoclonal antibodies (mAbs) with similar reactivity. Antibodies submitted to the workshop were coded and sent to participating laboratories which were requested to carry out immunohistological, immunocytochemical or FACS analysis on a range of tissues or cell lines. Data from these studies was used to perform a computerised cluster analysis. The results show that methods developed by FACS analysis of mAbs to leucocyte antigens can be applied to solid tumours. We have detected a cluster of 12 mAbs reacting with SCLC which also reacts with neuronal cells. Other smaller clusters of mAbs have also been detected. The method provides a basis for identifying and classifying antigens of any cell type and allows comparisons of different antibodies to be made in an unbiased fashion. In relation to small cell carcinoma of the lung, it has identified mAbs which have sufficient specificity to be useful in the classification of lung tumours and might have a future role in diagnosis or therapy.

**Dynamics of antibody transport and internalisation**

F. Matzku

*Institute of Nuclear Medicine, German Cancer Research Centre, Heidelberg, FRG.*

Antibody transport into target tissues and cells is determined by the biology of the antigen and by the structure of the MAb. Experiments using radiolabelled MAbs were carried out with cultured melanoma, lymphoma and sarcoma cells, and with athymic or competent animals bearing human or rodent tumour transplants. Biodistribution studies were done by immunoscintigraphy and whole body autoradiography. Internalisation into cultured cells was observed with 3/15 MAbs, which, according to metabolic requirements and the influence of bivalency, followed at least two different mechanisms. Complete internalisation of any type of externally bound MAb would invariably be achieved by anti-IgG antibodies. Yet, induced and 'spontaneous' internalisation were directed into different cellular compartments. *In vivo* accumulation in tumours depended on rapid clearance, as achievable with fragments. With an anti-lymphoma MAb, biodistribution was greatly influenced by the fact that antigen-positive normal and malignant cells showed functionally different internalisation *in vitro* and *in vivo*. Penetration into solid tissue was very limited with intact IgG, large areas of the nodes being left cold, but was gradually facilitated with fragments. Surprisingly, accumulation in haematogenous or lymphatic metastases was inversely correlated to size, suggesting preferential accumulation in nascent processes with putatively better blood supply.

**Cloning the cDNA for the core protein of a breast cancer associated mucin recognised by antibodies to the milk fat globule**

S.J. Gendler, J. Burchell, T. Duhig, A. Girling, R. Millis & J. Taylor-Papadimitriou

*Imperial Cancer Research Fund, PO Box 123, London WC2A 3PX, UK.*

Human mammary epithelial cells secrete and express on their surfaces, complex mucin glycoproteins of molecular weight >250 kd. Attention has recently been focused on these molecules, since many of the antibodies (among them HMFG1 and 2) selected for their reaction with differentiation or tumour-associated molecules, react differently with epitopes carried on these mucins. To study the regulation and analyse their molecular nature, we undertook to clone the gene for the core protein including SM-3, to select 7 cross-reacting clones from an expression library made from the breast cancer cell line MCF-7. Tissue specific expression of the gene is confirmed by the observation that mRNA is detected in breast epithelial cells but not in fibroblasts, carcinosarcoma or lymphoblastoid lines. Restriction enzyme analysis of human genomic DNA reveals extensive polymorphism which correlates with the polymorphism observed at the protein level. The availability of a cDNA coding for an epithelial-specific and tumour-associated mucin core protein will enable us to predict structure and to study the regulation of this molecule which is implicated in malignancy by virtue of its expression in nearly all breast tumours.

**Development and characterisation or monoclonal antibodies to the core protein of tumour associated mucins**

J. Burchell, S. Gendler, A. Girling & J. Taylor-Papadimitriou

*Imperial Cancer Research Fund, PO Box 123, London WC2A 3PX, UK.*

The most highly immogenic component of the human milk fat globule membrane is a large molecular weight mucin (>400 kd) which is also secreted into human milk. Furthermore, many of the tumour associated antigens, defined by monoclonal antibodies (MAb), are carried on molecules that are at the very least immunologically related to the milk mucin. Indirect evidence following the reaction of a MAB, HMFG2, with breast cancer cell lines suggests that the mucin produced by cancer cells may be aberrantly glycosylated. To explore this possibility, we have purified the milk mucin, deglycosylated it and produced MAbs to the mucin protein core. One of these antibodies, SM-3, reacts with 91% of breast carcinomas. Preliminary results show that it also reacts with carcinomas derived from other tissues. However, SM-3 shows little or no reactivity on pregnant and lactating breast, normal resting breast and benign breast tumours. It appears that this MAb is reacting with a protein determinant which is masked in normal cells but which is exposed, perhaps due to aberrant glycosylation in tumour cells. SM-3 and other MAbs to the mucin core protein have also been used to screen gt11 expression library and cDNA clones coding for part of a mucin gene.

**Use of monoclonal antibody 47D10 to monitor circulating tumour antigens**

M.K. Ho, W.P. Carney, H. Rabin & M. Sullivan

*E.I. du Pont de Nemours and Company, No. Billerica, MA 01862, USA.*

A mouse monoclonal antibody (MAb) 47D10 was produced against the human lung carcinoma line, A549. Immunohistochemical studies showed that MAb 47D10 was reactive with 57–95% of carcinomas derived from lung, breast, colon, pancreas. Staining was most intense with tissues from pancreatic and lung tumours. The antigen defined by MAb 47D10 was a glycoprotein of 67–97 kd on the surface of A549 cells. The extracellular form of the antigen showed a lower molecular weight of 60–80 kd. To determine whether the 47D10 antigen was shed into the circulation by tumours *in vivo*, a competitive ELISA was used to examine a panel of >260 serum samples. Circulating 47D10 antigens were found in sera of patients bearing breast, colon, pancreatic, ovarian and lung tumours. Even though immunohistochemical studies indicated strong reactivity of 47D10 MAb with lung carcinoma, relatively low levels of circulating antigen were detected in sera from patients with lung tumours. In contrast, >75% of sera from patients with pancreatic and ovarian tumours showed elevated levels of 47D10 antigens as compared to normal controls. Sera from patients with breast carcinoma could be differentiated from those with breast fibrocystic disease with a sensitivity of 70% and specificity of 88%. Furthermore, colorectal cancers with hepatic metastases showed significantly ($P<0.0001$) higher levels of 47D10 antigens than those with no hepatic involvement. Therefore, quantitation of circulating 47D10 antigens may be useful for monitoring of patients with pancreatic, ovarian, breast and colorectal cancers.

**Construction and expression of a tumour specific murine/human chimeric antibody.**

G.P. Moore

*Integrated Genetics Inc., Framingham, MA, USA.*

Variable region exons were cloned from heavy and light chain Ig genes of a murine hybridoma which secretes a human tumour-specific monoclonal antibody. These were fused to human R and L1 constant region exons and transfected into myeloma cells which secreted the resultant chimeric antibody. A series of experiments demonstrated that the chimeric antibody retained specificity for tumour antigen while acquiring various human characteristics. Moreover, the chimeric antibody was immunologically distinct from the murine MAb. Production of the chimeric antibody in myelomas was amplified 25-fold by cloning the fusion genes adjacent to the gene coding for dihydrofolate reductase and selection in methotrexate. The chimeric antibody was shown to bind to human tumours implanted in mice with pharmacokinetics identical to those of the murine monoclonal antibody. These results suggest that genetically engineered monoclonal antibodies may, in the future, be clinically useful replacements for Igs produced by murine hybridomas.

**Human monoclonal antibodies to colonic antigens**

C. Yiu, L. Baker, C.G. Clark and M.J. O'Hare

*Department of Surgery, University College London, WC1E 6JJ; Institute of Cancer Research, Royal Marsden Hospital, Sutton, Surrey SM2 5PX, UK.*

Despite much effort over the past 5 years, there remain difficulties in obtaining human monoclonal antibodies of pre-defined specificity. These include both low hybrid yields and hybrid instability as well as poor tumour localisation *in vivo*. Thus, collectively only about 100 human antibodies with defined reactivities to antigens of all types have been reported to date. In parallel with studies on a rat colonic tumour model, we have attempted to identify human antibodies from fusions of lymph nodes from patients with colorectal cancer and patients with inflammatory bowel conditions. We have used both our own HMy2 (Edwards *et al.*, *Eur. J. Immunol.*, **12:** 641, 1982) and the UC-729-6 (Glassy *et al.*, *Proc. Natl Acad. Sci. USA*, **80:** 6327, 1983) cell lines. We have also derived a variant which can be used to generate human hybridomas in high yield under serum-free conditions from the outset. However, attempts to increase hybrid yields with electrofusion techniques were unsuccessful. A total of 1,500 human hybrids have been prepared from 45 patients, but only 6 react strongly with colorectal tumour membrane preparations.

**Expression of functional mouse antibody directed against hPLAP in non-lymphoid cells**

V. Feys, P. de Waele, A. Van de Voorde, P. Casneuf & W. Fiers

*Laboratory of Molecular Biology, State University of Ghent, 9000 Ghent, Belgium.*

A mouse hybridoma cell line was isolated producing monoclonal antibodies (IgG2b, K) against the tumour-associated marker human placental alkaline phosphatase (hPLAP). The mRNAs coding for the heavy and light chains were cloned as cDNA copies. Detailed restriction analysis and nucleotide sequencing confirmed the IgG2B isotype. These genes were then separately inserted into the eukaryotic expression vector pSVd2-3tss+, a derivative of pSV-BG, under control of the SV40 early promoter. We obtained expression of a functional anti-hPLAP mouse antibody in a COS transient expression system and in a CHO permanent expression system. Both genes were introduced with the calcium phosphate technique in COS1 cells, and 72 h after transfection, $10\,ng\,ml^{-1}$ functional antibodies could be detected in the supernatant. Western blot analysis revealed that the molecular weight was identical with that of the mouse antibody isolated from the hybridoma cell line. Permanent CHO cell lines secreting $100\,ng\,ml^{-1}$ functional antibodies were established upon transfection of CHO (dhfr-) cells with the plasmids containing the H and L cDNAs and the plasmid pAdD26SV-(A)-3 carrying the mouse dihydrofolate reductase (*dhfr*) gene. These results indicate that not only lymphoid cells, but also non-lymphoid cells, are capable of synthesis and assembly of immunoglobulin chains that are immunologically competent. A plasmid construction in which we inserted a stop codon-containing sequence immediately following the hinge region of the H-chain cDNA sequence yielded immunocompetent $F(ab')_2$ molecules upon transfection of COS or CHO cells.

**Developments in radiochemistry**

C.F. Meares, S.V. Deshpande, S.J. DeNardo & D.A. Goodwin

*Departments of Chemistry and Nuclear Medicine, University of California, Davis, CA 95616, USA.*

The attachment of radioactive metals to monoclonal antibodies, for use in the diagnosis and treatment of cancer, is an exciting development. A problem that applies particularly to antibody-chelate conjugates is the accumulation of radioactivity in the liver. Using a monoclonal antibody against indium-L-benzyl-EDTA chelated, we have shown that practically all of the radioactive indium which is passed through the liver and into the urine remains in the form of indium benzyl EDTA chelated. Further, we have found that at least the majority of the radioactive indium remaining in the liver is still chelated to benzyl EDTA residues. This indicates that it may be possible to use metabolically cleavable linkages between antibody and chelate in order to reduce liver activity. We have compared linkages consisting of (1) a simple disulfide, (2) a simple diester, and (3) the peptide $(Ala\text{-}Leu)_2$-Gly. These three linkers lead to quite different organ distribution and clearance of indium-111 in mice. For the disulfide linkage, indium is rapidly translocated to the intestines; for the ester linkage, part of the radioactivity rapidly clears through the urine in the first 24 h, but the concentration of indium in the liver at 24 h is not greatly reduced; for the peptide linkage, clearance is slow and high levels of indium in the liver are observed. An alternative approach to reducing liver levels involves administering the indium-111 chelate in low molecular weight form, after a pre-targetting treatment with an antibody or other protein which can bind the small indium chelate. In this case very low liver levels may be obtained.

**Advances in radioimmunolocalisation with pre-clinical and clinical applications**

D.J. Hnatowich, F. Virzi & M. Rusckowski

*Department of Nuclear Medicine, University of Massachusetts Medical Center, Worcester, MA, USA.*

Clinical and pre-clinical studies using radiolabelled antibodies are currently in progress at the University of Massachusetts. In one investigation, the pharmacokinetics of the 19-9, OC-125 and 10-3D2 antibodies (Centocor Corp) radiolabelled with In-111 for imaging is being measured in patients. Despite identical protocols, significant differences in *in vivo* behaviour have been observed among these antibodies. For example, liver levels of In-111 following administration of labelled 19-9 were almost twice that of OC-125 and whereas these levels were constant throughout for both antibodies, in the case of 10-3D2, liver levels decreased uniformly. A second clinical investigation has determined the biodistribution in patients of OC-125 radiolabelled with Y-90, a pure beta emitter. Five patients undergoing second-look surgery for ovarian cancer were administered a tracer dose of labelled antibody i.p. and tissue samples obtained for counting. The results show that bone marrow will be the dose limiting organ but since tumour/normal tissue ratios of up to 25 were observed, it is likely that therapeutic efficacy will be ultimately established in subsequent high dose studies. Finally, the *in vivo* use of avidin and biotin is under consideration for imaging and therapeutic applications as a means of improving the localisation of radioactivity in the target. Antibodies have been conjugated with avidin and administered to animals before the administration of DTPA-coupled biotin radiolabelled with In-111. Using a model in which the target consisted of conjugated beads deposited in the peritoneum of mice, it has been shown that target:non-target radioactivity ratios may be significantly increased compared to conventional procedures.

**Progress and problems of radioimmunolocalisation**

J.F. Chatal

*INSERM U211, Nantes, France.*

Progress in immunoscintigraphy depends on several immunological, radiochemical and methodological variables. The criteria of antibody efficiency include strong affinity, recognition of a great number of antigens sites per target cell and good immunohistochemically-assessed specificity. An anti-GD2 antibody (produced by Cheung, Cleveland, USA) meets this criteria. Initial immunoscintigraphic results after radioiodination have been excellent, with contrasts which appear to be markedly greater than with other currently used antibodies. The choice of radionuclide is also important. After an initial phase of clinical studies with iodine-131, the tendency now in antibody labelling, is to use indium-111, which is more stable *in vitro* and *in vivo*. Comparative distributions in patients with tumours show increased tumour uptake and higher tumour-to-tissue ratios with indium-111. However, the high liver uptake hinders interpretation of upper abdominal images. Finally, regardless of the radionuclide used, the limiting factor of immunoscintigraphy remains the tumour-to-nontumour ratio which is usually too low to provide good scintigraphic contrast. Among the methods capable to improving contrast, SPECT is probably the best. Its use in comparison with that of planar scintigraphy has not yet been clearly demonstrated and comparative clinical studies are under way to determine the roles of the two detection methods. In terms of its clinical value, immunoscintigraphy has already shown its complementarity to other conventional diagnostic methods.

**Correlation of vascular permeability (VP) and tumour blood flow (BR) with antibody uptake in a renal cell carcinoma (RCC) xenograft**

H. Sands, P.L. Jones, S.A. Shah, D. Palme & R.L. Vessella

*Dupont, N. Billerica, MA; University of Minnesota, USA.*

The specific uptake of 125-I-A6H antibody by xenografts of the RCC in the athymic mouse was considerably greater than that seen for other human tumour xenografts and their associated antibodies. The A6H-RCC model also demonstrated both greater localisation indices and absolute amount of antibody bound than did the B6.2-Clouser model. Several physiological factors were studied to assess whether they might play a role in this greater specific uptake. VP was determined by measuring the amount of 125-I-BSA and 131-I nonspecific IgCl extravasated out of the vasculature during one hour. BF to the tumour was determined using the 86-Rb method. BF and VP were found to be greater in the RCC xenografts than in CL tumours. Differences in VP were dramatic, showing the vasculature of the RCC xenograft was twice as permeable as that of the CL tumour. Animals bearing RCC or CL xenografts were injected with a monoclonal antibody to human major histocompatibility complex (125-I-anti-HLA). Tumour uptake of 125-I-anti-HLA was found to be 5× greater in RCC than CL xenografts. These results, therefore, suggest that the differences seen in the physiological factors can account for some of the greater specific 125-I-A6H uptake by the RCC.

**Therapeutic use of radiolabelled monoclonal antibodies by systemic and locoregional administration**

P. Riva, G. Paganelli, G. Moscatelli, S. Benini

*1st Oncol Romanagnolo – Nuclear Medicine Department, M. Bufalini Hospital, Cesena, Italy.*

Eleven patients (2 breast, 2 lung, 3 colon, 4 ovary tumours), with advanced disease, were selected for therapeutic application of I-131-labelled MOAbs following failure of chemotherapy or radio-therapy. We used antibodies HMFG1, HMFG2, PLAP, AUA1 (kindly supplied by Dr Epenetos – London), F023C5 (Sorin Biomedica – Italy), and 494/32 (Behring – RFG). Antibody doses ranged between 10 and 30 mg, and the I-131 dose ranged between 50 and 150 mC. In 3 cases the MOAbs were administered i.v., whilst in 8 patients, the i.p. or i.pl. route was selected. No side effects were observed. All patients developed antimouse antibodies. The effective half-life of administered radioantibodies ranged between 30 to 70 h. The calculated dose delivered to tumour was 18–52 Gy. The clinical results, at present (follow-up between 3 and 18 months) can be summarised as follows: 1 complete remission, 3 partial remissions, 4 static disease and 3 progressive diseases. These therapeutical trials appear promising and suggest a more systematic use of this treatment in order to collect more conclusive data.

**Preparation and biodistribution of Indium-111 labelled chelate immunoconjugates of a monoclonal antibody to carcinoembryonic antigen**

D.K. Johnson, D.A. Westerberg, P.L. Carney, G.A. Sumerdo, P.E. Rodgers & S.J. Kline

*Abbott Laboratories, Abbott Park, IL 60064, USA.*

Novel bifunctional derivatives of the chelating agents ethylenediaminetetraacetic acid (EDTA) and diethylenetriaminepentacetic acid (DTPA), in which a 4-isothiocyanatobenzyl moiety is attached at a methylene carbon atom of one carboxymethyl arm of the chelator, have been synthesised. These derivatives were used to prepare immunoconjugates of an IgG1 monoclonal antibody to carcinoembryonic antigen (CEA). The resulting reagents showed no aggregation and no loss of immunoreactivity relative to the underivatized antibody, at substitution levels as high as 10 chelators per antibody molecule. Incubation for 30 min at room temperature with commercially available sources of indium-111 chloride consistently resulted in >95% radiochemical yields of labelled antibody, averting the need for any post-labelling purification step. Biodistribution studies in nude mice ($n=5$ per group) bearing a CEA-positive xenograft (LS174T), revealed comparable tumour uptake for both the EDTA and DTPA conjugates at 48 h post-injection ($20.9 \pm 4.2\%$ ID $g^{-1}$ and $19.7 \pm 5.3\%$ ID $g^{-1}$ respectively) but a significantly lower liver uptake from the DTPA conjugate ($3.9 \pm 0.8\%$ ID $g^{-1}$) compared to the analogous EDTA conjugate ($9.1 \pm 3.8\%$ ID $g^{-1}$, $P<0.05$ by Student *t*-test). Liver uptake at 48 h post-injection in non-tumour bearing Sprague-Dawley rats was also low for both conjugates, with no significant differences seen between the EDTA conjugate ($0.96 \pm 0.12\%$ ID $g^{-1}$) and its DTPA analogue ($1.07 \pm 0.27\%$ ID $g^{-1}$). We conclude that the characteristics of these chelator immunoconjugates, both *in vitro* and in animal models, are sufficiently encouraging to warrant clinical trials of their utility in radioimmunoscintigraphic detection of CEA-producing tumours.

### *In vivo* labelling of biotinylated anti-CEA monoclonal antibodies by radioactive avidin

G. Paganelli, P. Riva, G. Deleide, A. Clivio, F. Chiolerio, M. Malcovati & A.G. Siccardi

*Ospedale Bufalini, Cesena, Sorin Biomedica, Saluggia, SPA Milano, University of Milan, Italy.*

Several tumour associated monoclonal antibodies persist on the surface of tumour cells *in vivo*, with the best tumour/background ratios being achieved 72–96 h post-injection of radiolabelled Mab. We have developed a strategy to target nuclides to tumour bound antibodies, at a time when non-specific localisation has already been cleared, using subsequent administrations of biotinylated Mab and of nuclide-conjugated avidin. Preliminary experiments have been performed in rabbits. Native avidin labelled with 131-I and 111-In (as DPTA conjugate) was tested for its reactivity with biotin and was found to be between 93–100%. Biodistribution studies after both i.v. and i.p. administrations of the iodinated reagent showed a biological half-life of 24 h. The In-labelled reagent showed marked liver accumulation, possibly due to indium transferrin exchange. Biotinylated and non-bitinylated nitrocellulose targets were implanted in the peritoneal cavity of 5 rabbits. Radioactive avidin, administered i.p., accumulated on the biotinylated target at least 10 times more efficiently than on the control target.

### Labelling platelets with an indium labelled monoclonal antibody

A.M. Peters, I. Loutfi, P. Lumley, N. Courtenay-Luck & J.P. Lavender

*Hammersmith Hospital, London and Glaxo Group Research, Ware, UK.*

We have evaluated a new technique for labelling platelets using In-111-labelled monoclonal, IgG1, antibody (P256) raised against the platelet surface glycoprotein, GIIbIIIa (Imperial Cancer Research Fund, London). P256 was labelled by coupling to DTPA followed by chelation of In-111. In-111 uptake was 5–7 mCi $mg^{-1}$ P256. Platelet function following exposure to P256 was tested by measurement of spontaneous aggregation and aggregometry using a whole-blood platelet counter (Ultra-flow 199). A platelet receptor occupancy of 6% was the maximum that resulted in spontaneous aggregation in platelet-rich plasma (PRP), comparable to that seen in the absence of P256. The antibody can be shown to label platelets both *in vitro* and *in vivo*. The FAB/2 fragment of P256 has also been shown to label platelets *in vitro*. One normal volunteer and 12 patients with suspected venous thrombus have been imaged with either Indium-111 labelled P256 or its fragment. Localisation in thrombus was seen in 5. This technique therefore promises to be a simple specific method of platelet labelling.

### Imaging of colon cancer with I-131 human monoclonal IgM

J.A. Carrasquillo, R. Steis, M. Bookman, J. Smith, Del Vecchio, J. Reynolds, P. Perentesis, R. McCabe, M.V. Haspel, M. Hanna, D. Longo & S.M. Larson

*DNM, CC, NIH, Bethesda and BRMP, DCT, NCI, Frederick and Bionetics Research, Rockville, MD, USA.*

We have evaluated 2 different I-131 labelled IgM human monoclonal antibodies (HMoAb) for their ability to image tumours and their pharmacokinetics when given in escalating doses in 11 pts with metastatic colon cancer. Seven pts received 5 mCi and 8–108 mg of HMoAb 28A32 obtained from a human–mouse hetero-hybridoma, and 4 pts received 5 mCi and 8–58 mg of HMoAb 16.88 obtained from a transformed human lymphocyte. Both HMoAb react with a colon carcinoma associated antigen. All pts had positive immunohistology in a previously resected tumour biopsy. The antibodies were labelled via the iodogen method at a mean specific activity of 1.1 mCi $mg^{-1}$ with good retention of immunoreactivity. All pts underwent an initial study with 8 mg of HMoAb followed by a second study at the higher HMoAb dose 7 days later. Imaging was seen in 6/7 pts receiving 28A32 and in 3/4 pts receiving 16.88. Initially tumours appeared as 'cold' lesions with optimal contrast between tumour and non-tumour after 5 days that persisted up to 23 days. There was 29–38% of the dose in plasma at 24 h and a whole body clearance (T 1/2) of 1.5 days. No dose dependent differences were observed in the pharmacokinetics or tumour imaging. One patient had a positive skin test to 28A32 (but not 16.88) prior to receiving any HMoAb and was excluded from imaging. No definite antibody associated toxicity was observed. HMoAb IgM can be radiolabelled with I-131 and used safely and successfully to image sites of metastatic colon cancer.

### Tumour imaging of colorectal carcinoma with an anti-CEA monoclonal antibody

M. Gasparini, M. Ripamonti, E. Seregni & G.L. Buraggi

*Divisions of Nuclear Medicine and Experimental Oncology E, Instituto Nazionale Tumori, Milan, Italy.*

The 131-I-labelled anti-CEA monoclonal antibody (F023C5 Sorin Biomedica) was used in a preclinical *in vivo* study in an animal model and in a clinical study in patients with cancer. The results obtained with nude mice bearing a CEA-producing colon carcinoma showed the antibody's specificity, and defined parameters for imaging in relation to the dose and administration route. Initially, we performed a pilot study at the National Cancer Institute of Milan on 48 patients with colorectal carcinoma. High sensitivity (>90%) and high specificity were obtained in detecting local relapse and abdominal metastases. These data were confirmed in a multicentre trial organised by the Italian National Research Council on 300 patients with 700 tumour sites. We conclude that this method may be of clinical value.

### CA-125 and screening for ovarian cancer: Serum levels in 1,010 apparently healthy postmenopausal women

I.J. Jacobs, J. Bridges, I. Stabile, P. Kemsley, C. Reynolds & D.H. Oram

*Department of Obstetrics and Gynaecology, The London Hospital, Whitechapel, London, UK.*

One thousand and ten apparently healthy postmenopausal female volunteers over 45 years of age and with a history of amenorrhoea for greater than 12 months attended for venopuncture and pelvic examination. Serum samples were assayed using the Abbott Laboratories CA-125 radioimmunoassay kit. The 90th, 95th and 98th centiles for serum CA-125 in this group of postmenopausal women were 23.2, 26.3 and 30.85 U ml$^{-1}$ respectively. Thirty-one women with CA-125 level >30 U ml$^{-1}$ proceeded to ultrasound scanning. Three of these women underwent surgery following an abnormal ultrasound scan of which 1 had no abnormality, 1 a fimbrial cyst and the third a stage IB ovarian cancer. The other 28 had no abnormality on ultrasound scan and have been followed up with 3 monthly serum CA-125 measurement and repeat scans. In addition, 7 benign tumours found on vaginal examination and confirmed by ultrasound scans were removed at laparotomy. None of these 7 women had a serum CA-125 >30 U ml$^{-1}$.

We conclude that CA-125 has high specificity for ovarian cancer when used to screen postmenopausal women. To achieve maximal sensitivity the upper normal limit for serum CA-125 in a screening programme for ovarian cancer incorporating the CA-125 assay and ultrasound scanning should be set at 23 U ml$^{-1}$. This would result in ~10% of the screened population requiring an ultrasound scan. The value of the CA-125 assay for early diagnosis of ovarian cancer warrants further investigation in a randomised controlled study.

### A phase I study using pan T lymphocyte-ricin A chain immunotoxin to treat steroid resistant graft *versus* host disease (GVHD)

V. Byers, N. Kernan, J. Henslee, R. Gingrich, B. Blazer, R. Mischak, J. Kersey, R. O'Reilly, J. Thompson & P. Scannon

*Xoma Corp., Berkeley, CA; Sloan Kettering Institute, NY; U Kentucky; U Iowa and I. Minn., USA*

A phase I clinical trial has been completed in which bone marrow transplant recipients with grade II–IV steroid resistant GVHD were treated with an immunotoxin constructed by linking monoclonal antibody XMMLY-H65, directed against the CD5 antigen on human lymphocytes, to ricin A chain (XMMLY-H65). Patients received up to 14 daily infusions of XMMLY-H65 in escalating doses. To date, more than 14 evaluable patients have been accrued. The treatment was well tolerated without noticeable toxicity. The most consistent finding was a rapid drop in T-lymphocytes, which usually persisted throughout the course of infusions. Target tissue clearing was seen early with skin, during the first 2–5 days of infusion, and later with gut, within the first week after completion of infusions. Peripheral T-cells reappeared 1–3 weeks after treatment, without recurrence of GVHD. Anti-immunotoxin antibodies were present in some patients. We conclude that immunotoxin treatment represents a promising therapy for patients with severe acute or chronic GVHD.

### Studies with antibody/drug conjugates

T.F. Bumol

*Lilly Research Laboratories, Indianapolis, IN, USA*

Murine monoclonal antibody KS1/4, which recognises an epithelial/epithelial malignancy-associated antigen, has been utilised as a targetting vehicle with covalently attached vinca alkaloid moieties for the preclinical development of a site-directed tumour therapy strategy. Two conjugates; KS1/4-desacetylvinblastine (KS1/4-DAVLB, Lilly Serial £LY256787) and KS1/4-desacetylvinblastine-hydrazide (KS1/4-DAVLB-Hydrazide, Lilly Serial £LY203725) are currently being considered as clinical trial candidates. These agents represent different conjugation chemistry strategies and both demonstrate *in vivo* efficacy in a number of preclinical studies with human colon and lung adenocarcinoma xenografts. Both agents display activity in models with low tumour burden (90–100% tumour growth suppression can be achieved), establishes tumours (measurable tumour regressions) and experimental metastases (2–5× increase in mean survival time) with therapeutic index advantages over conventional chemotherapy. Toxicology experiments in rats and primates document that repetitive dose schedules can be tolerated and that both conjugates are significantly less toxic than corresponding dosages of free vinca alkaloids. KS1/4-DAVLB and KS1/4DAVLB-hydrazide represent high and low dose strategies for the application of these agents to human oncology.

### Clinical trials of immunotoxins for the treatment of solid tumours

L.S. Spitler

*Xoma Corp., Berkeley, CA 94710, USA.*

We have initiated clinical trials of monoclonal antibody ricin A chain immunotoxins XOMAZYME-MEL for therapy of metastatic melanoma and XOMAZYME-COL for therapy of metastatic colorectal cancer. A phase I trial of the antimelanoma immunotoxin in 22 patients has been completed. The side effects were related to the dose of immunotoxin and were generally transient and reversible. These included (1) a transient fall in serum albumin with an associated weight gain, and oedema without proteinuria; (2) malaise fatigue, myalgia, anorexia and fever; (3) a transient decrease in voltage on electrocardiograms without clinical symptoms, change in serial echocardiograms, or elevation of creatine phosphokinase MB isozyme levels. Symptoms due to allergic reactions were observed in 3 patients. There was one complete response, lasting 26 months after a single course of immunotoxin, and evidence of biological activity of the immunotoxin in 9 additional patients. Localisation of antibody and A chain to sites of metastatic disease was demonstrated. A phase II multicentre trial of the antimelanoma immunotoxin was conducted in 46 patients. Thirty-five received a 5 day course and 7 patients received 3 or 4 day courses (total = 42 patients). Review of data indicates that the side effects were similar to those observed in the phase I trial. Efficacy is also similar. Out of the 43 patients there were 3 partial responses and evidence of biologic activity in 10. Side effects in the ongoing Phase I trial of biologic activity in 10. Side effects in the ongoing Phase I trial of colorectal cancer are similar to those observed in the melanoma trial suggesting that they are due to the A chain rather than the antibody.

### Immunological tailoring of monoclonal antibodies (MAb) suited for immunotherapy of pancreatic carcinoma

K. Bosslet, H.U. Schorlemmer, G. Schulz & H.H. Sedlacek.

*Research Lab of Behrwingwerke AG, D-3550 Marburg/Lahn, FRG.*

Four mechanisms may be involved in the potential action of monoclonal antibody guided immunotherapy of pancreatic carcinoma.

(*a*) MAb dependent cellular tumour cytotoxicity (ADCC).
(*b*) MAb dependent complement mediated cytolysis.
(*c*) MAb induced anti anti idiotypes.
(*d*) MAb mediated inhibition of pancreatic carcinoma cell functions like endocytosis, lysosomal enzyme and superoxide anion secretion.

Mechanisms (*a*) and (*b*) are mediated by the isotype of the MAb molecule whereas (*c*) and (*d*) are induced by the idiotype. MAb BW 494 of IgG1 isotype is able to mediate functions described under (*a*) and (*d*). Using the sib selection procedure combined with a highly sensitive ELISA for the detection of spontaneous changes in the isotype from IgG1–IgG2a, we were able to establish a panel of isotype switch variants of MAb BW 494. The IgG2a switch variants had the same epitope specificity as the parental IgG1 MAb, but learnt to perform human complement mediated cytolysis with human pancreatic carcinoma cell lines very efficiently. Clinical trials to evaluate whether MAb BW IgG2a might be superior to MAb BW IgG1 in the immunotherapeutic treatment of human pancreatic carcinomas are in progress.

### Immunotherapy of pancreatic cancer with monoclonal antibody 494/32

G. Schulz, R. Klapdor, M. Bulcher, R. Kubel, K.H. Muhrer, H.P. Harthus, N. Madry & K. Bosslet

*Res. Dept, Behringwerke AG, 3550 Marburg; Dept of Int. Medicine, Univ. Hamburg; Dept of Surgery, Univ. Ulm; Dept Surgery, Univ. Gieben, FRG.*

The murine monoclonal antibody (MAb) 494/32 (BI 51.011) directed against a pancreatic cancer associated glycoprotein antigen was selected for a phase I/II therapy trial because of cytotoxic (ADCC) *in vitro* killing of these tumour cells, and inhibition of pancreatic cancer xenografts growth in nude mice. Fifteen patients with advanced pancreatic cancer received increasing doses of MAb (5–100 mg) over a time period from 5 to 10 days. In a second phase, 10 additional patients were treated with an additional dose of 100 mg MAb followed by up to 13 daily infusions with 30 mg MAb (highest cumulative dose 490 mg). During this treatment, serum levels of murine IgG were raised to 43.3 $\mu g\,ml^{-1}$. The serum half life of murine IgG ranged from 3–4 days. Repeated injections of high amounts of murine MAb were well tolerated when given within the first 15 days. Most patients (13/15) developed at that time, anti-murine antibodies which resulted in anaphylactic reactions when therapy was continued (1 patient) or when it was repeated after some weeks (3 patients). Two patients developed fatigue and a neuritis-like syndrome, 1 week after the last IgG infusion which resolved spontaneously by the next day. In some patients it could be shown that the anti-murine response was, in part, anti-idiotypic. Fifteen out of 25 patients were eligible for evaluation of therapeutic efficacy. Two patients (13.3%) responded with tumour regression documented by reduction of liver metastases and primary tumour as assessed by CT scan. This correlated with a decrease of tumour markers (CA 19-9, CEA) and clinical improvement. One of these patients is now in remission for 36 weeks. Four (26.6%) additional patients with progressive disease stabilised. Ten out of 15 patients are still alive. The median survival of all patients after diagnosis is 43 weeks.

### Dosimetry for radiolabelled antibodies – macro or micro?

M.J. Myers

*Hammersmith Hospital, London W12 0HS, UK.*

Dosimetric calculations for the therapeutic use of radiolabelled antibodies have usually involved macroscopic (so-called MIRD formulation) assumptions about the distribution of energy deposited within a tumour or organ. This type of calculation probably suffices for gamma and for moderately energetic beta radiation such as that from I-131 or Y-90. When less conventional radionuclides, such as Alpha and Auger electron emitters, are proposed as antibody labels and significant amounts of emissions with ranges in tissue of the order of individual cell dimensions can be deposited at the cell surface then a different sort of calculation, microdosimetry, must be performed. This is because the macro assumptions about the average amount of energy deposited in each small amount of tissue no longer hold. Practical dosimetry will probably involve both approaches with macrodosimetry used to assess risk to normal organs and microdosimetry for assessing potential damage to targets. Promises of specific antibody targetting at the individual cell must, however, be translated into reality for developments in microdosimetry to be applicable.

### Quantitative SPECT in nuclear medicine. Application in dose planning for radionuclide therapy.

S.E. Strand & M. Ljungberg

*Radiation Physics Department, University of Lund, Lund, Sweden.*

In nuclear medicine imaging, SPECT is being used with increasing frequency for routine studies of different organs. A new approach is to use quantitative SPECT for dose planning in radionuclide therapy. Proper dose planning requires an absolute qualification of the activity of the therapeutic radiopharmaceutical uptake in different tissues. In order to calculate the absorbed dose, the specific activity ($MBq\,g^{-1}$) must be determined with high accuracy. Using SPECT, the specific activity can be determined if the activity in, and the volume of, the target can be estimated. An essential parameter in SPECT images to consider is the photon attenuation. A new method for attenuation corection based on measured attenuation charts of the actual object has been developed. Using the attenuation chart, the emission image is corrected pixel by pixel for photon attenuation. With this method, it is possible to reduce the margin of error due to attenuation to less than e.g. 5% for 140 KeV photons. By using quantitative SPECT with attenuation correction, for dose planning in radionuclide therapy, it is possible to calculate the absorbed dose to the target volume, with a margin of error of $<10\%$.

### Quantitation in I-131 radioimmunotherapy using SPECT

S.J. Riggs, A.J. Green, H.J. Begent & K.D. Bagshawe

*Cancer Research Campaign Laboratories, Dept of Medical Oncology, Charing Cross and Westminster Medical School, London W6 8RF, UK.*

In radioimmunotherapy (RIT), antitumour antibodies are used to deliver I-131 specifically to the tumour. For the effects of RIT to be clearly understood, it is necessary to quantify the concentrations of the radionuclide in various organs. Planar gamma camera imaging has been used in previous studies but this leads to problems where there are overlying organs or tissue. SPECT overcomes these problems by providing a 3-dimensional representation of the activity distribution. Planar imaging and SPECT were compared in phantom studies and in patients receiving therapeutic doses of I-131-labelled anti-CEA. The method of Thomas *et al.* (*Radiol.*, **133,** 465, 1979) was used for quantitation. From the planar images for SPECT, back projection reconstruction was used with attenuation correction. Scattered photons were compensated for using data acquired simultaneously from a secondary window. All data were obtained on an IGE Gemini 700 gamma camera with a high resolution 400 KeV collimator. The limitations of SPECT quantitation were examined using phantom studies and it was shown that practical SPECT quantitation can be achieved in patients given therapeutic doses of I-131-labelled anti-CEA. This is likely to be of value in dosimetry for RIT and other treatments using I-131.

**Treatment of human tumour xenografts with I-131-labelled monoclonal antibodies**

R. Senekowitsch, H. Glassner, G. Reidel, G. Mollenstadt, H. Kriegel & H.W. Pabst

*Department of Nuclear Medicine, TU and Department of Nuclear Biology, GSF, Munich, FRG.*

An iodinated monoclonal antibody (MAb) with a high tumour deposition of $>50\%$ $IDg^{-1}$ and a long tumour residence time with a half-life of 5 days was used for treatment of human mammary tumour xenografts in nude mice. The radiation dose received by the tumour xenografts after injection of $2\times7.4$ MBq of the tumour specific MAb was calculated as 50 Gy, by sequential scintigraphic follow up of the injected animals. The therapeutic effect of the activity resulted in a pronounced reduction of the tumour diameter compared to animals injected with the same amount of unlabelled MAb, I-131-labelled non-specific MAb or only saline. On histological examination of the tumour tissue, large areas of necrosis and a high amount of pyknotic cells could be detected. In some animals the remaining tissue in the tumour at day 42 after injection could be identified as only mouse connective tissue invading the human tumour xenografts.

**Advances in Neuro-Oncology**

H. Coakham

*Brain Tumour Laboratory, Department of Neurological Surgery, Frenchay Hospital, Bristol, UK.*

Selected panels of MCAs recognising neuroectodermal antigens, intermediate filament proteins, epithelial and leucocyte antigens have been used in immunohistochemistry to increase the diagnostic accuracy of brain tumours by 20% in over 250 cases. Similarly, accurate diagnosis of tumour cells in cerebrospinal fluid (CSF) has been achieved in 95% of cases, resulting in important changes in management.

*In vitro* binding assay on tumour homogenates with 131-I-specific and 125-I non-specific MCA show high 'dose delivery' and specificity. However, i.v. injections produce scintigraphic tumour images but result in low specificity and indices and $<0.005\%$ injected dose delivered to tumour.

In contrast, administration of labelled MCA into CSF pathways produces more specific tumour binding. Biological responses and increased survival (1–2 years) were seen in 5/7 patients given antibody-guided therapy (11–50 mCi-131-I) for neoplastic meningitis from medulloblastoma, lymphoma, melanoma and pineoblastoma. All cases had relapsed after optimum conventional therapy. No significant side effects occurred. This approach may offer an alternative to the prophylactic external beam radiation which is used to achieve CSF 'clean up' in certain paediatric malignancies but has adverse effects on CNS development.

**Antibody guided diagnosis and therapy**

A.A. Epenetos

*On behalf of Imperial Cancer Research Fund Oncology Group, Royal Postgraduate Medical School, Hammersmith Hospital, London W12 0HS; Imperial Cancer Research Fund Laboratories, London WC2, UK.*

Progress is continuously being made in the *in vivo* applications of monoclonal antibodies, with the introduction of new pharmaceuticals, detailed pharmacokinetic studies and evaluation of techniques for tumour imaging. Based on our experience, we now recommend that for meaningful radioimmunolocalisation, one should incorporate specific and non-specific antibodies for tumour imaging.

Thirty-four patients with resistant ovarian cancer have been treated with i.p. $^{131}$I-labelled antibodies (HMFG1, HMFG2, AUA1, H17E2). There were no significant responses in 7 patients with gross disease. There were 2 responses in 13 assessable patients with tumour nodules of 2 cm in diameter. Out of 6 patients with microscopic disease, 5 are disease free with target follow-up being over 3 years.

A new phase I-II has been initiated using $^{90}$Y as radiolabel. Clinical pharmacokinetic studies have been performed and encouraging responses with no toxicity have already been observed. This new method of treatment appears to be of value in patients with residual ovarian cancer.

**Human immune responses: The full network**

M. Ritter, N. Courtenay-Luck, G. Sivolapenko & A.A. Epenetos

*Departments of Clinical Oncology and Immunology, Royal Postgraduate Medical School, Hammersmith Hospital, London W12 0HS, UK.*

Both patients and healthy controls have low serum levels of IgM antibody rheumatoid factors which react with both human and murine IgG1. These rheumatoid factors cause the pre-existing human anti-mouse response.

After one therapeutic administrstion of murine antibodies, patients develop antibodies which react with the Fc portion of the murine immunoglobulin. Subsequent administrations lead to the generation of both anti Fc and anti $F(ab')_2$ antibodies. A component of the anti-$F(ab')_2$ response is anti-paratopic. These anti-paratopic or anti-idiotypic antibodies (anti-id) reflect the internal image of tumour associated antigens and in *in vitro* assays, block the binding of the administered murine antibody to its antigen.

In those patients with anti-id$^1$ antibodies, antibodies with binding specificities similar to the administered murine antibodies are detectable. These are anti-id$^2$ or anti-tumour antibodies generated by either an anti-idiotypic network or by elevated circulating antigen.

### Anti-ovarian carcinoma anti-T3 heteroconjugates or hybrid antibodies induce tumour cell lysis by cytotoxic T-cells

S. Canevari, S. Menard, D. Mezzanzanica, S. Miotti, S.M. Pupa, A. Lanzavecchia & M.I. Colnaghi

*Instituto Nazional Tumori, Milan, Italy and Institute for Immunology, Basel, Switzerland.*

In the perspective of therapeutic *in vivo* targetting for T-cell attack, the monoclonal antibody (MAB) MOv18, selected for its restricted reactivity with human ovarian carcinoma and an anti-T3 MAB, were used to produce heteroconjugates or hybrid antibodies derived by the fusion of the relevant hybridomas. The specificity and the activity of the bispecific MABs were analysed by solid-phase RIA, immunofluorescence, competitive binding and a 51-Cr release assay on the ovarian carcinoma cell line OVCA432 which expresses the relevant tumour-associated antigen and on several irrelevant tumour cell lines and normal cells. Both reagents efficiently promoted, at nanomolar concentration, target cell lysis by cytotoxic T-cell (CTL) clones. Although the pattern of the tumour cell lines which were lysed was wider than that predicted by the binding studies, further studies using a more sensitive biochemical technique confirmed that the specificity of the MABs is superior to heteroaggregates both in purification recovery and storage stability. Peripheral blood lymphocytes could also be used as cytolytic effectors, provided that a suitable *in vitro* activation scheme was adopted.

### Tumour therapy with vinca alkaloids targetted by a hybrid–hybrid monoclonal antibody recognising both CEA and vinca alkaloids

J.R.F. Corvalan, W. Smith & V.A. Gore

*Lilly Research Centre Limited, Eli Lilly and Co., Erl Wood Manor, Windlesham, Surrey, UK.*

The functional properties of a hybrid-hybrid monoclonal antibody recognising both CEA and vinca alkaloids have been explored *in vivo*, in nude mice xenografted with MAWI, a human colorectal tumour. The hybrid-hybrid monoclonal localises specifically onto CEA expressing tumour tissue and, furthermore, is able to target vinca alkaloids to it. Under the influence of the hybrid-hybrid monoclonal, a profound change in the biodistribution patterns of the vinca alkaloids are observed. Therapeutic data produced in this *in vivo* model indicated that treatment with vinca alkaloids in conjuction with hybrid-hybrid monoclonal antibody is significantly more effective in suppressing tumour growth of established tumour xenografts than the alkaloids given as free drugs.

### Intraperitoneal injection of In-111-labelled monoclonal antibodies in a nude mouse model intraperitoneally grafted with a human ovarian cacrinoma

P. Thedrez, M. Kremer, C. Curtet, S. Pelhate, C. Sai Maurel, J.C. Saccavini, D. Guerreau & J.F. Chatal

*INSERM U211, Nantes, France.*

The purpose of this work was to study the biodistribution of several indium-111-labelled monoclonal antibodies with known affinity for ovarian carcinomas in a nude mouse model i.p. grafted with a human ovarian cancer (NIH: OVCAR3). An immunohistochemical (immunoperoxidase) study compared the reactivity of 3 antibodies – DF3, OC125 and HMFG2 – for the NIH: OVCAR3 cell line, by computing an index of antigenic expression which took into account the percentage of stained cells and the intensity of staining. For the 3 antibodies, this index was respectively 166, 114 and 73. Nude mice were injected i.p. with $2 \times 10^7$ cells, and the peritoneal cavity became progressively lined by tumour nodules before the animals died at $80 \pm 5$ days. The first biodistribution studies were performed with In-111-DTPA-OC125 antibody (In-OC125) injected i.p. in intact form 50–60 days after i.p. grafting of the tumour cells. The animals were sacrificed 2 and 24 h after injection (5 mice for each time period), and the results expressed as percentage of injected dose $g^{-1}$ (% ID $g^{-1}$) and in tumour-to-organ ratios. A nonspecific immunoglobulin labelled under the same conditions (In-NS) was injected into the same number of mice. With In-OC125 the % ID $g^{-1}$ in the tumour was $14.4 \pm 0.7$ and $27.8 \pm 8.6$ at 2 and 24 h respectively; with In-NS the percentage for the same time periods was $8.9 \pm 1.7$ and $4.4 \pm 0.8$. With In-OC125 the tumour/blood, tumour/liver and tumour/kidney ratios were respectively $3.7 \pm 0.8$, $4.8 \pm 0.9$ and $3.7 \pm 0.9$ at 24 h; with In-NS the same ratios were $1.1 \pm 0.3$, $1.2 \pm 0.7$ and $0.7 \pm 0.2$. These first results are very encouraging and suggest that the same distribution study should be carried out for therapeutic purposes by replacing indium-111 with yttrium-90.

### Absolute uptake of I-131 HMFG2 monoclonal antibody administered intraperitoneally in malignant and benign ovarian tumours

J. Malamitsi, D. Skarlos, G. Aravantinos, P. Papakostas, S. Vassilarou, A. Varvarigou, S. Fotiou, A.A. Epenetos & C. Koutoulidis

*NIMTS Hosp., Dimocritos, Athens, Greece.*

Eight patients, 5 with known ovarian cancer (OC) and 3 with pelvic masses proven afterwards to be benign, underwent immunoscintigraphy with I-131 HMFG2. After i.p. administration of 500–1500 $\mu$Ci I-131 HMFG2, biodistribution of the antibody was studied. Biopsy material was taken on a laparotomy and counted in a $\gamma$-counter.

*RESULTS*

| *Pt. no.* | *Disease* | *Immunoscan* | *Absolute uptake % adm. dose g$^{-1}$* |
|---|---|---|---|
| 1 | OC peritoneal metastases | + | 0.1 |
| 2 | OC peritoneal metastases | + | 0.13 |
| 3 | OC retroperitoneal metastases | – | 0.0019 |
| 4 | OC peritoneal metastases-ascites | + | 0.0008 (0.0275 ml$^{-1}$, ascites) |
| 5 | OC complete remission | – | 0.0017 |
| 6 | serous cyst | – | 0.0011 |
| 7 | dermoid cyst | – | 0.0032 |
| 8 | serous cyst | – | 0.0016 |

In pts. 1 and 2, proven positive, absolute uptake was 100 times higher than that in pt 5, who was in complete remission; 3, who had a single retroperitoneal metastasis and 6, 7 and 8, who had benign ovarian tumours. In pt 4, however, with a rapidly reaccumulating ascites, very low uptake was found in metastatic tissue, while higher uptake values were found in the ascitic fluid.

**Biodistribution, pharmacokinetics and imaging of I-131 labelled OC125 in patients wirh ovarian carcinoma**

H.J. Haisma, K.R. Moseley, A. Battaile, V.R. Zurawski & R.C. Knapp

*Department of Gynaecologic Oncology, Dana Farber Cancer Institute, Brigham and Woman's Hospital and Harvard Medical School, Boston, USA.*

Fifteen patients with or suspect of having ovarian carcinoma were injected i.v. or i.p. with 131-I labelled OC125 $F(ab')_2$. Immunoscintigraphy was performed at the time of injection and at 24, 48 and 72 h after injection. Blood and urine samples were used for pharmacokinetic studies. Biopsy specimens were obtained at laparotomy and uptake of antibody in selected tissue was calculated. Radioimmunoscintigraphy after i.v. injection revealed 50% of the tumour sites. After i.p. injection all tumour sites were visualised, except in one patient where the antibody stayed localised because of adhesions. One patient with endometrial cancer showed no specific uptake of the antibody after i.p. injection. The serum half life of the radiolabelled antibody after i.v. injection was 30 h. At 24 h after injection, 20% of the injected dose remained $l^{-1}$ blood. After i.p. injection, there was a slow appearance of radiolabelled antibody in the blood with a maximum level of 1.4% dose $l^{-1}$ at 24 h after injection. Urinary excretion of the radiolabel was the same for both routes of administration, with 50% of the dose excreted in 50 h. Tumour uptake was $2.3 \times 10^{-3}$% of injected dose $g^{-1}$ tissue after i.v. injection and $4.8 \times 10^{-3}$% of injected dose $g^{-1}$ tissue after i.p. injection. Liver and bone marrow uptake was $2 \times 10^{-3}$% injected dose $g^{-1}$ tissue after i.v. injection and $<1 \times 10^{-3}$% injected dose $g^{-1}$ after i.p. injection. I.p. administration of monoclonal antibodies in ovarian carcinoma patients is preferred to i.v. injection both for imaging and for specific uptake in tumour tissue.

**Suppression of the anti-mouse antibody response to a monoclonal antitumour antibody in rabbits with cyclosporin A**

J.A. Ledermann, H.J. Begent, J.A. Boden, F. Searle & K.D. Bagshawe

*Cancer Research Campaign Department of Medical Oncology, Charing Cross Hospital, London W6 8RF, UK.*

Single doses of radiolabelled antibody have not been effective in eradicating tumours, so it is likely that patients will require multiple doses for successful tumour therapy. However, this cannot usually be given as most patients develop anti-antibodies after one or two treatments. A method has been developed to suppress the anti-antibody response to repeated doses of a mouse monoclonal anti-human chorionic gonadotrophin antibody. Rabbits injected i.v. with 200 $\mu$g antibody were given cyclosporin A (CsA), 20 mg $kg^{-1}$, i.m. for 6 days starting at day 1. They were rechallenged with antibody after 14 days under cover of CsA. In some cases, antibody was ultracentrifuged to reduce the immunogenicity due to microaggregates. In the animals receiving CsA, the clearance of mouse antibody was significantly prolonged and the rabbit anti-mouse antibody response was absent in 8/8 given ultracentrifuged antibody and 6/8 given the standard preparation. Ultracentrifugation alone did not prevent the anti-antibody response. We are now giving patients CsA and ultracentrifuged antibody to prevent the formation of anti-antibodies to repeated doses of radiolabelled monoclonal anti-CEA antibody used for the therapy of advanced colorectal cancer.

### Abstracts of Poster Exhibits

**Monoclonal antibodies 123C3 and 123A8 identify a new neuroendocrine marker.**

D. Schol, S. Wagenaar & J. Hilgers

*Division of Tumour Biology, The Netherlands Cancer Institute, 1066 CX Amsterdam, The Netherlands.*

Monoclonal antibodies 123C3 and 123A8 were generated against a crude preparation of membranes from a small cell lung carcinoma (SCLC) specimen. Their tissue/tumour distribution was identical. Western blot analysis showed an antigenic molecule at 29 kd, under reducing conditions. Non reducing conditions revealed a band at $\sim$150 kd.

In normal tissues, the antigen is present in neural and neuroendocrine components like nerves, brain tissues and certain epithelial populations such as Islands of Langerhans and bronchial glands. In breast, thyroid and testis, positive cells are scattered over the entire epithelium. The tumour distribution also supports a putative neuroendocrine nature of the antigens. Positive tumours include SCLC (25/25), bronchial carcinoids (14/14); bronchial adenoid cystic carcinomas (5/5) and medullary thyroid carcinomas (3/3). Adenocarcinomas (26/27) and squamous cell carcinomas (50/58) of the lung, follicular thyroid carcinoma (1/1) and carcinomas of the breast (6/8) are negative.

Both antigens can be demonstrated on the plasma membrane of living cells. These data suggest that 123C3 and 123A8 define novel cell surface markers for neuroendocrine differentiation.

**Simultaneous demonstration of glia and glioma associated antigens in human astrocytomas**

T. Bilzer, D. Stavrou & B. Martin

*Institut fur Tropoyhologie, Veterinastr 13, 8 Munchen 22, FRG.*

Glia fibrillary acidic protein (GFAP) and glioma associated antigens (GAA) defined by monoclonal antibodies (McAbs) were demonstrated simultaneously in human astrocytoma tissue. GFAP was stained by PAP-method, GAA were visualised by ABC-technique using alkaline phosphatase.

Fresh tumour samples from biopsy material were immediately frozen in liquid nitrogen and propagated in tissue culture, respectively. Tumours were grafted to nude mice by inoculation of $5 \times 10^6$ cells and cryosections were then taken from primary and secondary tumours. For production of McAbs against GAA, BALB/c mice were immunized with whole glioma cells.

In both primary and secondary tumours, heterogeneity of GFAP- and GAA expression was obvious. While GFAP was restricted mostly to cell processes and less marked in the perinuclear space, McAb-positive material was located either in the tumour cell, surface membrane or cell processes. There was remarkable expression of GAA in cell clusters which failed to express GFAP. At higher magnifications, three types of cellular reactivity were detected; (a) cells reacting only with anti-GFAP, (b) cells reacting only with anti-GAA and (c) cells expressing both GFAP and GAA. Because these cells can be found in s.c. tumour grafts, they may represent not only reactive cells, but also be part of the tumour cell populations.

**Immunocytological localisation of cancer-associated antigens recognised by monoclonal antibodies using immunogold techniques**

R. Mandevile, M.G. Zelechowska, I. Ajdukovic, A. Amarouch, S. Ghali, A. Hill & B. Grouix

*Immunology Research Center, Institut Armand-Frappier University of Quebec, Laval-des-Rapides (Quebec), Canada.*

We have developed several monoclonal antibodies (MAbs) that show a different spectrum of reactivity towards normal and neoplastic breast cancer cells. These MAbs recognise breast cancer-associated antigens expressed, either exclusively (BCD-B4) or preferentially in the cytoplasm (BCD-E8) or on the cell surface membrane (BCD-F9) of breast carcinoma. Colloidal gold was utilised as a immunocytochemical marker both at the light microscopic level using thin silver-enhancement technique and at the electron microscope level using thin sections and a modified protein-A gold technique. Double labelling techniques using different size (10 nm and 2 nm) probes were also employed on the same section of breast tissue to simultaneously localise and compare the reactivity of 2 MAbs. These techniques allowed us to confirm our earlier immunohistochemical observations using direct immunofluorescence and ABC techniques and to further identify the subcellular localisation of our MAbs. We believe that this rapid and accurate technique might prove to be the method of choice for the visualisation of antigenic sites at the light and electron microscope levels using MAbs. Further development of these techniques may give further qualitative and quantitative information on the distribution of tumour-associated antigens within cells and tissues.

**Plasma fibrin degradation products as a marker of ovarian cancer**

E.P. Lauraine, M.C. Mirshahi, J. Soria, C. Soria, M. Mirshahi, R. Montalva, J.Y. Perrot, C. Boucheix, T. Darse & A. Bernadou

*Hotel Dieu, Paris (4); INSERM U150, Paris and INSERM U268 Villejuif, France.*

We examined the relationship between tumour proliferation and activation of blood coagulation products. Blood activation results in the formation of fibrin which is degraded by fibrinolysis producing fibrin degradation products (FDP). Using an ELISA assay with a monoclonal antibody specific for FDP, we studied FDP plasma concentrations in 50 patients with ovarian cancer.

In untreated patients, FDP levels correlated with abdominal tumour size as measured by laparotomy and CA125 assays. In 6/50 patients, however, only the FDP level reflected the abdominal spread and thus was the best marker with which to follow the evolution of these patients. On chemotherapy, two groups of patients were examined comparing FDP levels with those of CA125. In group 1 ($n=14$) FDP levels acted as a tumour marker, since a good correlation was found between the level of FDP and that of CA125 and tumour size. In group 2 ($n=10$), FDP levels remained high, despite a significant decrease in CA125 levels and in tumour volume. The ratio FDP/CA125 was at least 5 times higher than the initial ratio. Chemotherapy had a similar toxicity in the 2 groups, but the response evaluated by a second laparotomy was better in the patients of group 2 than in those of group 1 (complete remission was never observed in the 14 patients of group 1, but was observed for 6/10 patients in group 2). Therefore, high levels of FDP in patients on chemotherapy is associated with a better clinical response. We hypothesise that, in group 2 patients, stimulation of monocytes or macrophages by chemotherapy (causing elevated FDP levels), may explain the better response to chemotherapy.

**The use of CA125 as a tumour marker for adenocarcinomas of endocervix, endometrium and fallopian tube**

A. Scharl, G. Crombach, H. Musch, M. Vierbuchen & A. Bolte

*Department of Obstetrics and Gynaecology, Institute of Pathology, University of Cologne, FRG.*

CA125 is a common constituent of the glandular epithelium of the female genital tract. The clinical significance of CA125 as a tumour marker in carcinomas of the endocervix, endometrium and fallopian tube was tested by immunohistochemical detection of CA125 in 54 cancer tissues and by radioimmunometric measurement of CA125 levels in 12 cancer cytosols and in serum samples of 60 patients with recurrent carcinomas. Positive immunohistochemical staining was present in 80% of the adenocarcinomas. In well differentiated tumours the CA125 antigen was mainly concentrated at the apical poles of the cytoplasms and in the luminal mucus. In poorly differentiated carcinomas a predominantly diffuse cytoplasmic staining was observed. At least 35% of the cancer cells in each tissue section were negative for CA125. In cancer cytosols of the endometrium, the CA125 levels ranged from 173 to 13,215 U $mg^{-1}$ cell protein. The CA125 serum concentrations were below the cut-off value of 65 U $ml^{-1}$ in 85% of patients with primary cancer and in cases with positive immunohistochemical staining and high cytosol levels. Elevated serum levels were mainly observed in patients with advanced primary cancer (stage III/IV; 30%) and recurrent carcinomas (79%). Apparently, the release of the antigen into the circulation is prevented by a tissue-blood-barrier, which has to be penetrated by a substantial quantity of infiltrative tumour tissue and/or vessel invasion. Therefore CA125 may be a useful tumour marker only in advanced carcinomas of the endocervix, endometrium and fallopian tube.

**Increased detection of micrometastases in the bone marrow of patients with small cell lung cancer (SCLC)**

F. Hay, A. Ford & R. Leonard

*Imperial Cancer Research Fund Medical Oncology Unit, Department of Haematology, University Department of Clinical Oncology, Western General Hospital, Edinburgh EH4 2XU, UK.*

Rapid and accurate detection of metastases in the bone marrow of patients with SCLC may have therapeutic implications. Analysis of bone marrow is done routinely by conventional histological examination of marrow smears which has a good detection rate where foci of tumour cells are present or large numbers of cells infiltrate the marrow. It is less satisfactory where single cells are sparsely distributed throughout the marrow. We report on a series of 18 bone marrows from patients with SCLC that were assessed for tumour infiltration in several ways – routine histology; cytospin preparations of red cell depleted marrow with immunohistology using 2 MoAbs (HMFG2, CAM5.2); detailed examination of multiple air dried preparations of red cell depleted bone marrow with a panel of 10 SCLC-associated MoAbs and capacity to grow in serum-free defined media (Hites). In 4/18 cases, the routine marrow examination revealed tumour infiltration and in 6/18 of the cytospin preparations tumour cells were seen. Using the larger panel of MoAbs a further 9 cases were thought suspicious of tumour involvement although only isolated cells were detected. In 8/9 of these we sustained growth of cells morphologically similar to the putative tumour infiltrate in Hites medium for periods of 1–18 weeks, 2 remain viable as established cell lines. These results indicate that detailed analysis of bone marrow using a panel of MoAbs may be worthwhile particularly in programmes utilising autologous marrow rescue as intensification therapy.

### Rhythmic oscillations of CEA levels in patients with breast cancer

W. Jager, L. Wildt & W. Sauerbrei

*Department of Gynaecology and Obstetrics, University of Erlangen, FRG.*

Previous studies using polyclonal anti-CEA antibodies demonstrated considerable undulations of CEA serum levels during a 24 h period in patients with metastatic breast cancer. This phenomenon was re-examined with an immunoradiometric assay applying 3 monoclonal anti-CEA antibodies (CEA-ELISA, ID-CIS, Dreireich, FRG) for measurement of the same serum samples. The coefficient of variation for duplicate determinations in that assay system was always below 5%. Hourly serum samples for CEA determination had been obtained from 9 patients suffering from metastatic breast cancer. Irrespective of the assay, the re-appearing fluctuations of CEA serum levels could not be explained by the variations caused by the assay. They seem to be an inherent phenomenon of CEA secretion in that disease. Peak concentrations could be detected every 4 to 6 h. These rhythmic oscillations seem to be independent of cortisol and prolactin levels, as well as of sleeping periods. The comparison of monoclonal and polyclonal measured CEA concentrations revealed parallel as well as opposing courses, which might be explained by the heterogeneity of CEA. Further studies should be done to evaluate if these rhythmic oscillations are a unique property of CEA release from cancer cells or a consequence of impaired metabolism and clearance.

### Demonstration of estramustine-binding protein in human lung cancer cell lines using immunohistochemical and fast protein liquid chromatography techniques

J. Bergh, J.E. Westlin, O. Brodin, P.A. Bjork & S. Nilsson

*Department of Oncology, University of Uppsala, Akademiska Sjukhuset, S-751 85 and LEO Research Laboratories, S-214 00 Helsingborg, Sweden.*

The cytostatic drug estramustine used in the management of prostatic carcinoma, binds *in vivo* to an intracellular protein, the estramustine-binding protein (EMBP). This protein has been previously isolated and characterised from rat ventral prostate, and has also been discovered in human normal and malignant prostatic tissues. In this study, we describe the presence of EMBP in human lung cancer cell lines.

A panel of well characterised human lung cancer cell lines was used, representing both the small (SCC) and non-SCC group. A monoclonal antibody raised against the binding protein was applied on frozen sections using the avidin-biotin-peroxidase-antiperoxidase technique.

EMBP was demonstrated in 6/6 non-SCC cell lines. There was heterogeneity and variability within the majority of these cells. One important exception, the SCC cell line U-1906, was homogeneously stained. The other three SCC cell lines were negative. Furthermore, we have confirmed the presence of EMBP in human lung cancer cell lines by fast protein liquid chromatography.

These results form a basis for extended studies on biopsies and on cell lines to clarify the mechanism of action of this drug.

### Selection of anti-carcinoembryonic antigen monoclonal antibodies suitable for immunoscintigraphy and therapy

P. Seguin, J.C. Saccavini, B. Darbouret, F. Degorce, J. Sertour & E.J.P. Jolu.

*Compagnie Oris Industrie, BP 121, 91190 Gif sur Yvette, France.*

It is well known that monoclonal antibodies raised against carcinoembryonic antigen (CEA) often cross react with other related molecules such as NCA I and NCA II. Antibodies intended for *in vivo* use should have high affinity and specificity for CEA.

We have investigated 12 anti-CEA Mabs in order to select those suitable for immunoscintigraphy and therapy. All Mabs were IgG1 and the affinity constants ranged from $10^8$ to $2.10^9\,l\,M^{-1}$. Epitopes on the CEA molecule were determined by binding inhibition of 125-I-labelled CEA on coated Mabs. The antigenic mapping showed 6 different epitopes.

The cross reactions of the Mabs with the NCA molecules (50 kd and 95 kd) were tested using an $^{125}$I-labelled antigen preparation. Six Mabs showd no cross reactions: these results were confirmed by binding of $^{125}$I-labelled Mabs on isolated peripheral blood granulocytes.

Immunoperoxidase was carried out on a panel of normal frozen tissues: colon, liver, pancreas, lung, skin, thymus and tonsil. Five Mabs corresponding to 3 epitopes showed a strict specificity. Most cross reactions involved granulocytes. One Mab stained biliary ducts.

After iodination, Mabs were tested for their immunoreactivity: they were submitted to an immunoaffinity assay using CEA coupled to Sepharose. The *in vivo* performances of 3 Mabs recognising different epitopes on the CEA molecule were compared in tumour bearing nude mice. The immunoaffinity assay on CEA-sepharose was found to be a good way to select Mabs suitable for *in vivo* use.

### CA50. A useful tumour marker in monitoring patients with colorectal cancer

B.E. Persson & J. Holmgren

*Surgical Clinic 1, Sahlgeen' Hospital and Department of Medical Microbiology, University of Goteborg, Goteborg, Sweden.*

Using a monoclonal antibody-based radioimmunoassay inhibition method, we have determined pre-operative serum levels of the carcinoma associated carbohydrate antigen CA50 in 266 patients with primary colorectal cancer. CA50 levels exceeding the mean value for blood donor sera by more than 2 standard deviations were found in 47% of these patients. 15%, 43%, 31% and 65% were elevated in patients with Dukes A, B, C and D respectively. Only 5% of patients with benign colorectal disease had elevated levels and these were patients with ulcerative colitis of a duration of $>10$ yr. Among patients who had developed a known recurrence after operation for a primary Dukes' A–C colorectal cancer, 66% had elevated levels, 25% of resected patients with no clinical evidence of disease after operation also had elevated CA50 levels. From 139 patients operated for colorectal cancer a definitive rise in CA50 was demonstrated in 12

cases. Clinical evidence of recurrence developed in these cases with lead times of CA50 titre rises ranging from 5–40 months. Our finding suggest that a rise in CA50 levels after resection of a Dukes' A–C primary colorectal cancer is indicative of a recurrence and may preceed clinical evidence of disease by several months or even years. Thus CA50 may be a useful tool for monitoring patients with colorectal cancer.

### Evaluation of tumour markers CA19-9, CA125 and CEA in pancreatic cancer and other diseases

H.Y. Zhang

*Department of Nuclear Medicine, Zhong Shan Hospital, Shanghai Medical University, Shanghai, China.*

We performed simultaneous measurements of tumour markers CA19-9, CA125 and CEA. The results were as follows:

| | *Frequency of positive results* (%) | | |
|---|---|---|---|
| | *CA19-9* | *CA125* | *CEA* |
| A. Malignant disease: | | | |
| pancreatic cancer | 23/25 (92.0) | 10/25 (40.0) | 12/25 (48.0) |
| colorectal cancer | 12/61 (19.6) | 8/61 (13.1) | 16/61 (26.2) |
| gastric cancer | 10/40 (25.0) | 4/40 (10.0) | 11/40 (27.5) |
| cholangiocarcinoma | 8/11 (72.7) | 3/11 (27.2) | 4/11 (36.3) |
| breast cancer | 0/23 (0) | 5/23 (21.7) | 4/23 (19.3) |
| B. Benign disease: | | | |
| pancreatic disease | 2/22 (9.0) | 3/22 (13.6) | 1/22 (4.3) |
| gastrointestinal disease | 2/38 (5.2) | 4/38 (10.5) | 11/38 (28.9) |
| cholangeitis | 6/20 (30.0) | 3/20 (15.0) | 4/20 (20.0) |

Our results confirm previous studies indicating that CA19-9 may be a useful marker for monitoring pancreatic carcinoma.

### Elimination of fibroblasts from mixed cultures of neoplastic origin

T.W. Briers, T.M. Vandeputte, P.M. Stroobants, E.J. Nouwen, R.L. Marynissen & M.E. De Broe

*Department of Nephrology-Hypertension, University Hospital, Antwerp, Belgium.*

The elimination of human fibroblasts from primary cultures continues to be a major drawback in the use of *in vitro* techniques. Most known methods, for the elimination of normal proliferating fibroblasts from a mixed culture, e.g. trypsinization, quick passage and mechanical elimination are unsatisfactory. Our goal was to develop methods for the elimination of fibroblasts, that are fast and reliable. We selected a mixed cell culture of a permanent fibroblast cell line, FLOW-4000 (human embryonic kidney, Flow Laboratories, 02-010) and HeLa-H21 cell line which expresses alkaline phosphatase. The expression of alkaline phosphatase was used to calculate the ratio of HeLa-H21 cells over the total cell population. Using this model system we tested several products that are supposed to reduce or eliminate fibroblasts in mixed cultures: primary culture plates (Becton Dickinson, 3847), putrescine (Janssen Chimica, 23-1400-1), D-valine medium (Gibco, 041-2570), cytosine arabinoside (Janssen Chimica 22.718.20), and genticin (Sigman, G5013). Out of this series, genticin was the only product that was able to selectively eliminate fibroblasts out of mixed cultures.

### Evaluation of monoclonal antibody binding to human tumour cells in immunocompetent animals using diffusion chambers

J.G. Fjeld, O. Bruland, K. Nustad & H.B. Benestad

*The Norwegian Radium Hospital, Oslo, and Institute of Physiology, University of Oslo, Norway.*

Preclinical studies of labelled antibodies for tumour imaging or immunotherapy are usually performed with the nude mouse xenograft model. One main objection to this model is that when the MoAbs are given to mice they are allogenic, but xenogeneic when given to patients. Therefore, a novel method for preclinical MoAb evaluation which can be used in any animal species has been developed. In this model the tumour is an i.p. diffusion chamber (DC), either filled with cells or antigen-coated particles. The DC is made of 0.22 $\mu$m micropore membranes and heat sealed to both sides of a plastic ring. The membranes allow diffusion of molecules, while cells and particles are kept within the DC.

In the first experiments, normal BALB/c mice were used to compare our DC system with results from nude mice. The experiments were performed with the monoclonal H7 (a gift from T. Stigbrand) against placental alkaline phosphatase (PLALP) and two sarcoma-associated MoAbs (TP-1 and TP-3). The cell lines used were Hep2 (expressing PLALP), and the osteosarcoma cell line OHS. Each mouse received two DC, one filled with a suspension of OHS, and one filled with Hep2. After the implantation, the mice were randomised into groups, and each group was injected i.v. with one of the MoAbs (IgG or fragments) labelled with 125-I. Thereby, the two cell lines were mutually serving as antibody binding cells or controls. When intact IgGs were injected, radioactivity ratios (DC cell suspension/blood) of 3–8 were achieved. With Fab or $F(ab')_2$ fragments the ratios were higher, but the percentage of injected dose bound to the cell was lower.

### Preclinical *in vivo* study with an anti-tumour monoclonal antibody: Factors affecting radiolocalisation

M. Ripamonti, S. Canevari, M. Gadina, A. Turrin, M. Gasparini, G.L. Buraggi & M.I. Colnaghi

*Instituto Nazionale Tumoti, Milan, Italy.*

An 131-I-labelled anti-CEA monoclonal antibody (Sorin Biomedica F023C5), shown to detect neoplastic lesions by radioimmunoscintigraphy (RIS) was tested in an animal model prior to clinical therapeutic use. Nude mice bearing a CEA producing human colon carcinoma received 131-I-labelled anti-CEA $F(ab')_2$ fragments. Comparison between the RIS and specific activity (SA), i.e., the ratio of cpm $mg^{-1}$ in the tumour to the cpm $mg^{-1}$ in the blood, muscle and kidney, showed that: independently of tumour site (i.p. or s.c.) i.p. administered antibodies gave better SA than i.v. RIS was negative. This could be attributed to an instrumental limitation. Five per cent radioactivity remained in the tumour with the i.v. injection, whereas up to 20% radioactivity accumulated in the tumour and i.p. administration. Data on repeated injections indicate a rise in tumour uptake.

**_In vitro_ targetting of normal and neoplastic B- and T-lymphocytes using 111-Indium-labelled monoclonal antibodies**

I. Loutfi, R. Batchelor, J.P. Lavender & A.A. Epenetos

*Department of Immunology, Diagnostic Radiology and Clinical Oncology, Royal Postgraduate Medical School, Hammersmith Hospital, London W12 0HS, UK.*

Preliminary tests have been conducted *in vitro* using human T- and B-cell lines as well as whole blood, to establish the usefulness of two murine monoclonal antibodies, an anti-CD5 and a Pan B, for radioimmunolocalisation and therapy. Both monoclonal antibodies showed specificity for the cell lines in question as tested by indirect immunofluorescence. Radiobinding assays on cell lines and whole blood showed binding of the 111-In-labelled antibody on up to 40% of lymphocytes in whole blood. These results should permit successful *in vivo* targetting of normal and neoplastic B- and T-cells.

**Optimization of imaging in relation to diethylene triamine-pentaacetic acid anhydride (DTPA) labelling techniques**

D.E.A. Zanin, P.F. Bruning, J. Hilkens & P. Hol

*Netherlands Cancer Institute (Antoni van Leeuwenhoekhuis), Plesmanlaan 121, 1066 CX Amsterdam, The Netherlands.*

Two monoclonal anti-MAM6 antibodies, designated 115D8 (IgG2b) and 140C1 (IgG1) have been labelled with 111-In via DTPA-chelation and assessed for localisation in human ovarian carcinoma xenografts in nude mice.

DTPA was attached to the antibody in varying molar ratios of 4:1, 8:1, 16:1, in order to determine the influence of the DTPA-coupling on the *in vivo* distribution of the MoAb. Following removal of the free DTPA on a sephadex G25 column, 111-In-tropolonate was added to the DTPA-MoAb. Unbound 111-In was separated from the bound fraction using a sephadex G25 column. The specific radioactivity was kept the same in each study. Tumour bearing nude mice, with comparable tumours, were injected i.p. and imaged at several time intervals. The mice were then dissected and the organs weighed and counted for radioactivity. We found that superior images were obtained when low DTPA:MoAb ratios were used and thus these labelling conditions are recommended for clinical studies.

**New prospects in the treatment of hepatocellular carcinoma (HCC): 131-I lipidol, a promising agent?**

P. Bourguet, J.F. Bretagne, J.L. Raoul, R. Duvauferrier, S. Coornaert & J.Y. Herry

*CHU Pontchaillou, 35033 Rennes, France.*

The aim of this study was to assess the biodistribution and therapeutic benefit of 131-iodine labelled Lipiodol in patients with liver tumours.

*Patients and methods:* 42 patients aged 44–78 years, with liver cirrhosis (10), liver metastasis (13) or HCC (19), were investigated. Two ml of 131-I-labelled Lipiodol (70 MBq) were injected into the hepatic artery via a Seldinger's catheter. Scans were performed over the abdomen and the thorax, 6 h after injection and at days 4, 8, and 15. Eight patients with metastasis and 7 with HCC received, 2–4 weeks later, a second injection of 900–1600 MBq of I-131 Lipiodol.

*Biodistribution results:* (1) in patients with metastasis and HCC, there was no activity outside the liver and the lungs. (2) 64% to 91% (median 82%) of the injected dose was taken up by the liver after 6 h. (3) For the HCC, the cpm ratio of tumour to normal liver ranged from 2.4–6 (median 4.6). (4) The slopes of radioactivity decay were identical in the normal liver and the lungs, but smaller in the tumour. (5) The effective half life of 131-I Lipiodol ranged from 3.6–10 days (median 4.6). (6) During an 8 day sampling period, the iodine radioactivity in the urine was 30–50% (median 42%) of the injected dose. (7) Less than 1% of the injected dose was found in the stool.

*Therapeutic results:* Clinical improvement was noticed in all patients. A significant drop in serum levels of tumour markers (CEA, CA 19.9 or AFP) was observed (>25% after one week, >50% after 3 weeks); for the 3 patients with HCC who survived more than 4 weeks, a significant reduction in tumour size was observed.

**Colony growth of phytohaemagglutinin stimulated peripheral blood lymphocytes in patients with malignant melanoma**

Z. Rudolf, G. Krosl & G. Sersa

*The Institute of Oncology, Ljubljana, Yugoslavia.*

Lymphocytes from peripheral blood in patients with malignant melanoma were cultivated for 7 days using two-layered cultivation method in semisolid medium. The ability of PHA stimulated peripheral blood lymphocytes to form colonies was tested in 43 patients with malignant melanoma and 15 healthy donors. In patients with malignant melanoma a significantly lower colony count was observed when compared to healthy controls ($951 \pm 581$ *vs.* $1734 \pm 250$; $P<0.05$). The mean number of colonies in 10 patients with local recurrence ($575 \pm 267$) was lower than in 11 patients with good response to treatment ($1536 \pm 419$) ($P<0.05$). In 7 patients with disseminated disease, the mean value of colonies was $799 \pm 480$. A similar value was found in 10 patients receiving interferon treatment ($557 \pm 346$). Both groups were compared with a group of healthy donors and patients responding to treatment and the differences were found to be significant ($P<0.05$). In some patients the test was repeated at different times in the course of the disease. The number of colonies decreased with progression of the disease, while the opposite was observed when response to treatment was achieved.

**Hybridoma screening for monoclonal antibodies to soluble tumour antigens: Inadequacy of solid phase immunoassays**

U. Auerswald, H. Kalthoff, W. Schmiegel & H.G. Thiele

*Medizinische Klinik, Universitatskrankenhaus Hamburg-Eppendorf, FRG.*

Generation of monoclonal antibodies (mAbs) to human gastrointestinal tumours often yields mAbs with carcino-embryonic antigen (CEA) specificity. In an attempt to develop a rapid and reliable strategy for detecting CEA reacting hybridoma-supernatants (HS) at an early stage of the screening process, we tried 3 different approaches: (1) HS were screened by fluorescent immunoassay (FIA) with purified CEA coated onto plastic wells. Forty-eight HS from 2,000 tested, were positive. (2) Fifty-three HS were tested applying soluble, biotinylated CEA. Twenty-two were positive, 5 of which were negative in the solid phase assay. (3) Thirty-eight HS were tested in Western blot assay with nitrocellulose bound CEA after SDS-PAGE. Eleven HS showed a positive reaction, 8 of which had been positive in solid phase FIA and solution based assay, 3 of them only in solid phase assay. These data indicate that >50% of positive results obtained by solid phase immunoassays are false positive when re-examined in solution based assays. Yet the latter system may fail to detect specific antibodies in some cases. These findings suggest that a combination of different assays is necessary to determine antibody specificity.

### Monoclonal antibodies reacting with DMH induced rat colorectal cancer

L. Baker, C. Yiu, M.J. O'Hare, B.A. Gusterson, L. Borman & C.G. Clark

*Department of Surgery, University College London and Institute Cancer Research, Sutton, UK and University of Vermont, USA.*

The dimethylhydrazine (DMH) induced colorectal cancer in rat is a good model for the human disease. We have raised mouse monoclonal antibodies to this tumour for targetting studies. A crude membrane extract of the tumour from Wistar rats or RCC2, a cell line derived from a Fischer tumour transplant 4047 were used as immunogen. I.p. immunisations were performed weekly until a humoral response was detected. NSO parental cell was used as the fusion partner. Immunoperoxidase staining on frozen sections was used for antibody selection. Five antibodies were obtained with 6 Wistar fusions. These were Lab 27.19 (IgM), Lab 22.1, Lab 22.10, Lab 3.3 (IgG2a) and Lab 26.15. All antibodies react with the Wistar tumour and normal colon. Lab 27 also reacts with the kidney, pancreas and the Fischer transplant. The remaining antibodies react with most epithelial cells and some with red blood cells. Four were obtained with six RCC2 fusions. These were JB28.4 (IgG1), JB39.4 (IgG1), JB2.6 (IgG1) and JB7.4 (IgM). All antibodies react with the Fischer transplant, Wistar tumour and normal colon. JB28.4 also reacts with the pancreas and Fallopian tubes. The other antibodies react with various epithelial cells and some with erythrocytes. These 4 antibodies show positive immunofluorescence with live RCC2 cells. Iodinated JB28.4 binds with live RCC2 xells and *in vivo* tumour localisation studies in the Fischer rat are currently under way.

### Preparation and *in vivo* study of Yttrium-90 labelled immunoconjugates

D. Snook, G. Rowlinson & A.A. Epenetos

*Imperial Cancer Research Fund Oncology Group, Department of Clinical Oncology, Royal Postgraduate Medical School, Hammersmith Hospital, London W12 0HS, UK.*

90-Yttrium has been suggested as a suitable isotope for radioimmunotherapy. This pure beta emitter (max 2.3 MeV, t1/2 = 64 h) can be produced from a 90-Sr generator. The monoclonal antibody HMFG1 and its $F(ab')_2$ fragment were coupled to DTPA using the bicyclic anhydride method. The number of DTPA molecules per antibody was estimated and immunoreactivity was tested by ELISA. 90-Y was reacted with each conjugate at pH 6.0, 22°C for 2 h, giving specific activities of 0.8–1.3 $\mu Ci\,\mu g^{-1}$ (81–91% labelling efficiency). Immunoreactivity post-radiolabelling was assessed by ELISA and RIA. Athymic nude mice received i.p. 14 $\mu g$ of either HMFG1-DTPA-90-Y or HMFG1 $F(ab')_2$ DTPA-90-Y. Results were expressed as percentage injected dose $g^{-1}$ tissue (%ID $g^{-1}$). The blood activity peaked at 2 h (20% ID $g^{-1}$) for both conjugates with a t1/2 of 39 h and 14 h for HMFG1 and $F(ab')_2$ respectively. Over 3 days, liver, spleen and femur activity increased while lung activity decreased for both conjugates. Liver and femur activity were the same at 3 days (12% ID $g^{-1}$ for HMFG1, 10% ID $g^{-1}$ for $F(ab')_2$). The most significant differences were in kidney levels; 26% ID $g^{-1}$ for $F(ab')_2$ by 3 days (20% ID $g^{-1}$ at 5 days) compared with a constant 6% ID $g^{-1}$ for intact IgG. Free DTPA-90-Y injected i.p. was cleared via the kidneys to the urine within 2 h, as did free 90-Y but with some accumulation in bone (32% ID $g^{-1}$ at 3 days). I.v. injected 90-Y colloid accumulated in the liver (49% ID $g^{-1}$ at 24 h) while i.p. 90-Y colloid remained in the peritoneum. Kinetic studies such as these are a necessary prerequisite for selecting and optimising antibody-guided irradiation.

### MRI after injection of specific NMR contrast agent Gd-25 DTPA-MAb, in nude mice bearing human colon adenocarcinoma

C. Curtet, C. Bourgoin, J. Bohy, P. Thedrez & J.F. Chatal

*INSERM U211, Faculty of Medicine, Nantes; ORIS Industrie, CEA Saclay, France*

Monoclonal antibodies (MAbs) 19-9 and 73-3 specific for human colon adenocarcinoma were labelled with a high number of gadolinium atoms. Twenty-five DTPA were chelated per MAb, with only slight loss of immunoreactivity. The NMR contrast agent Gd-25, DTPA-MAb 19-9 or 73-3 ([Gd] 17 $\mu mol\,kg^{-1}$, [MAb] 60 $\mu m$) were injected into nude mice bearing human colon adenocarcinoma (SW 948 or HRT 18). Tumours were removed 24 h after injection and T1 was measured *in vitro* with Bruker Minispec 20 MHz. T1 relaxation time varied according to MAb specificity against tumour targets. T1 decreased 20% for MAb 19-9 and MAb 73-3 with SW948 tumour. Imaging was performed with this model on Bruker Mini-Imager 4.7 teslas (Spin-echo, short sequences, TR 500 ms, TE 30 ms). Good contrast was obtained 24 h after Gd-25 DTPA-MAb injection.

### Intraoperative tumour detection by radiolabelled antibodies

N. Pateisky, Ch. Schatten & K. Philipp

*1st Department Obstetrics and Gynecology, University of Vienna, A-1090, Austria.*

Although radioimmunoscintigraphy (RIS) has progressed rapidly over the last few years, tumours $<1$ cm in diameter cannot be diagnosed by RIS due to the limitations of scintigraphic imaging. In an attept to improve this, we developed a hand-held gamma ray detection probe for intraoperative application. With this device it is now possible to scan over the operation area during the surgical procedure. In this way, the probe can be brought into close contact with questionable malignant tissue. This should result in an enhanced sensitivity in the detection of small tumour masses in contrast to conventional RIS. The results achieved so far in a small number of patients ($n = 6$) bearing either colorectal or ovarian cancer confirmed this suggestion. Possible applications of this method are: Isotope-guided biopsies, assessment of completeness of resection, and detection of tumour sites $<1$ cm.

### Single photon emission computer tomography *versus* two dimensional imaging with radiolabelled MoAbs in patients with melanoma

D.E.A. Zanin, P.F. Bruning & J. Hilkens

*Netherlands Cancer Institute (Antoni van Leeuwenhoekhuis), Plesmanlaan 121, 1066 CX Amsterdam, The Netherlands.*

Thirteen patients with metastatic melanoma, were studied by (gamma camera) radioimmunoscintigraphy. All patients received i.v. 18–28 mCi $^{99m}$Tc-antimelanoma MoAb F(ab′)$_2$ and imaged after 6–8 h. Four patients were further studied at 18–24 h using Single Photon Emission Computer Tomography (SPECT). Scans were performed with a Siemens dualhead gamma camera. Both planar imaging and SPECT detected malignant lesions in 11/13 (84%) patients. In 4 patients we were able to detect lesions which were not found by conventional diagnostic procedures. These lesions were situated in the pelvis or abdomen and were confirmed by follow-up. However, not all known lesions were detected, especially small pulmonary metastases, because of high background activity in the cardiovascular system. The application of SPECT improved the anatomical localisation of tumour sites and gave a superior depth resolution resulting in more true positives (3/4). This preliminary study indicates that SPECT can improve the results of dimensional radiolabelled MoAb distribution within an organ, provided very careful quality controls are performed.

### The pre-operative detection of clinical and subclinical lymph node metastases in patients with breast cancer using 131-I-labelled HMFG2 and F(ab′)$_2$ fragments of HMFG1

A. Athanassiou, D. Pectasides, K. Pateniotis, L. Tzimis, A. Lafi, D. Maintas, J. Taylor-Papadimitriou, A. Cross & A.A. Epenetos

*Metaxas Memorial Hospital, Piraeus, Greece and Imperial Cancer Research Fund and Hammersmith Hospital, London, UK.*

In an attempt to pre-operatively detect clinical and subclinical axillary lymph node metastases in patients with breast cancer, we used the 131-I-labelled HMFG2 and F(ab′)$_2$ HMFG1 monoclonal antibodies. We studied 10 patients with clinically obvious axillary lymph node disease (group A), 10 patients with clinically negative axilla (group B), using 131-I-labelled HMFG2 and 5 patients with clinically negative axilla (group C), using 131-I-labelled F(ab′)$_2$ HMFG1. All patients had clinical diagnosis of breast cancer. Each patient received 1–1.5 mCi (specific activity 5 mCi mg$^{-1}$ protein) as a s.c. injection into the webs between the 2nd and the 3rd fingers of both hands. The healthy side was used as a control. The patients were scanned at 24 and 48 h after the injection. In group A, 7/10 patients had positive scans. Histology and immunoperoxidase staining confirmed the presence of tumour in the lymph nodes in all patients.

In group B there were 4 true positive scans, 4 true negative, 1 false positive (due to non-specific reaction) and 1 false negative. Histology and immunoperoxidase staining showed lymph node involvement in 5 patients. In group C there were 4 true negative scans and the histology confirmed the absence of tumour in the axillary lymph nodes. In one patient the radiolabelled antibody was arrested in the middle of the right arm probably due to lymphatic obstruction, although no clinically apparent lymphoedema was present.

These results indicate that this non-invasive approach can pre-operatively detect tumour in axillary lymph nodes with a high degree of accuracy and can be of value in the diagnosis and staging of patients with breast cancer.

### Monoclonal antibodies reactive with lung squamous cell carcinoma. Cell reactivity and scintigraphic trial

L. Dazord, D. Bourel, B. Collet, P. Bourguet, J.C. Saccavini, Ph. Delaval, B. Desrues & L. Toujas

*CRLCC, CTS, CHU, Rennes, France and CEA, Saclay, France*

Po22, Po43, Po60 and Po66 mouse monoclonal antibodies were produced by immunization against a human lung squamous cell carcinoma. The tissue reactivity of antibodies was monitored by immunoperoxidase staining of frozen or paraffin sections. The antigens were studied by immunoprecipitation and SDS gel electrophoresis. The antigen immunoprecipitated by Po43 and Po60 from lung squamous cell carcinoma appeared as a single band of a MW 70 kd. Po66 recognised an antigen with a relative MW 47–50 kd. Purified monoclonal antibody Po66 and an unrelated IgG1 and immunoglobulin were labelled with radioactive iodine and injected i.v. into nude mice bearing s.c. xenografts of lung squamous cell carcinoma. The localisation index in the tumour was 3.3. This allowed scintigraphic imaging of the xenografts which were clearly outlined by days 9–11. Clinical trials in progress suggest that the Po66 antibody will be suitable for tumour imaging.

### A controlled prospective immunoscintigraphic study in patients planned for continuous regional chemotherapy of liver metastases

R.P. Baum, M. Lorenz, C. Hottenrott, E. Staib-Sebler, G. Hib-nauer, J. Happ & G. Hor

*J.W. Goethe University Hospital, Frankfurt, FRG.*

Continuous hepatic artery infusion chemotherapy (CHAI) of liver metastases from colorectal cancer should only be performed in patients (pts) without extrahepatic tumour. However, the detection rate of small metastases outside the liver by conventional diagnostic procedures is low. Therefore, we used immunoscintigraphy (IS) in pts planned for CHAI in an attempt to increase diagnostic accuracy.

Seven pts were injected with 62 MBq I-131 labelled F(ab′)$_2$ fragments of MAb 19-9/anti-CEA. Computer acquisitions were recorded 2–13 days PI. IS was done prospectively (i.e. without clinical/radiological information) in 40 pts. The findings were compared to the distribution of disease at subsequent surgery (and with CT scan/sonography) and confirmed by histology and immunohistochemistry. Sensitivity of IS for liver metastases was 85%, for extrahepatic abdominal disease 80%, and for pelvic recurrences 91%. In 18%, IS was the only diagnostic method to detect extrahepatic tumour involvement. In 51% IS was complementary to CT/sonography. By immunoperoxidase staining, 79% of tumours ($n = 42$) were CEA, and 62% were CA19-9 positive. Serum levels of CEA and/or CA19-9 (RIA) were elevated in 82%.

We conclude that immunoscintigraphy can contribute useful information in the pre-operative staging of pts planned for regional chemotherapy.

### Squamous cell carcinoma (SCC)-antigen in diagnosis and therapy monitoring of cervical carcinoma

P. Stieber, P. Meier, W. Eiermann, B. Bayerl & A. Fateh-Moghadam

*Institute of Clin. Chem., Department Gynaecology Obstetrics, Klinikum Gorbhadern, University of Munich, FRG.*

SCC-antigen is a tumour antigen (glycoprotein, MW 48,000 Daltons) purified from squamous cell carcinoma of the uterine cervix. Preliminary results with a recently developed immunoradiometric assay (ABBOTT) for the determination of SCC in serum have shown it to be a good marker in monitoring therapy of patients with cervical squamous cell carcinoma. The assay proved to be linear over the whole range of the calibration curve up to 150 ng ml$^{-1}$. Analytical recovery yielded between 75% and 94%. The interassay co-efficient of variation (VK) at 3 different levels was between 6.4 and 12.7%. The reference range based on 35 individuals (20–51 years) in whom neither clinical signs nor laboratory results were suggestive of any disease, covered 0.1–1.5 ng ml$^{-1}$ (cut off value: 2.0 ng ml$^{-1}$; mean value: 0.61 ng ml$^{-1}$; median: 0.6 ng ml$^{-1}$). One out of 15 patients with benign gynaecological disease had a slightly increased SCC-level (CEA was below the cut-off level of 3 ng ml$^{-1}$). We determined SCC and CEA in 300 sera of 34 patients with cervical carcinoma. Twenty-one out of 34 patients showed elevated SCC-levels pre-operatively (74%), whereas CEA was only elevated in 29%. Within a group of patients with relapse or metastatic spread, we found in 71% increased SCC levels (CEA 42%). Only one patient showed an isolated elevated CEA value. Our results suggest that the SCC-RIA is a useful tool in diagnosis and monitoring therapy of cervical squamous cell carcinoma.

**Clinical experience with radioimmunodetection (RID) using anti-melanoma antibodies**

P. Oehr, A. Hotze, H. Biltz, W. Meinhof, U. Bull & H.J. Biersack

*Institute Nucl. Med., University Bonn and Department Nucl. Med., University of Aachen, FRG.*

Since 1985, the $^{99m}$Tc-labelled monoclonal anti-melanoma antibody (AB Technemab K, Sorin Biomedica, F(ab')$_2$ fragments against the surface antigen 225.28S), was used in RID studies. The present study reports results gained at two centres. RID was performed in pts either with metastases of a known primary lesion or in pts with unknown primary lesion but proven metastases. 350 $\mu$g of the antibody labelled with 740 MBq $^{99m}$Tc were injected. Imaging was performed on a large field gamma camera at various times from 1 h to 24 h. Fifty pts with a total of 103 known metastases were studied. These results of RID were then compared with those obtained by clinical course, ultrasound, CT and/or immunohistochemistry.

RESULTS:

| | *True pos.** | *True neg.* | *False pos.* | *False neg.* | *Total* |
|---|---|---|---|---|---|
| Pts [*n*] | 21 (42%) | 17 (34%) | 2 (4%) | 10 (20%) | 50 (100%) |
| Lesions [*n*] | 34 (33%) | 18 (18%) | 2 (2%) | 49 (48%) | 103 (100%) |

*pos: in a patient = at least 1 lesion detected.

| | *Sens.* | *Spec.* | *Pos. pred. val.* | *Acc.* | *Neg. pred. val.* | *Preval.* |
|---|---|---|---|---|---|---|
| Pts. | 68% | 89% | 91% | 76% | 63% | 62% |
| Lesions | 41% | 90% | 50% | 27% | 81% | 81% |

Immunohistochemistry was positive in 9 out of 10 cases studied. In 7 patients where the primary tumour was undetectable by all other means, RID was also negative. We conclude that in ~30% of the pts studied, RID enabled detection of hitherto unknown metastases.

**Absorbed dose in skin from contamination with $^{111}$In when labelling**

S.E. Strand, G. Grafstrom & B.A. Jonsson

*Radiation Physics Department, University of Lund, Lund, Sweden.*

The radioisotope $^{111}$In is used in labelling of leucocytes, granulocytes, platelets and antibodies. Because of its liquid state there is always a risk of contamination during the handling procedure; as the oxinate is soluble in fat, the contamination may penetrate the skin. Emission of low-energetic electrons during the decay would cause high absorbed dose. The aim of this investigation was to evaluate the 'normal' grade of contimination during labelling and the absorbed dose encountered.

The degree of contamination during different labelling procedures was investigated by determination of the activity on protection gloves used by personnel. Measurable activity remaining after all handling procedures ranged from 0.1 to 100 kBk.

Leakage in different latex protection gloves, with or without simulation of excessive sweating, showed fractions up to one percent of contaminated activity. Penetration in skin was evaluated with two different methods. Autoradiography and special measurements with a surface barrier detector showed that the main activity stays on or near the surface and not exceeding a depth of 10 $\mu$m.

**Immunoscintigraphy of tumours before and after radiotherapy**

P. Oehr, V. Bjorkland, B. Bjorkland, A. Bockisch & H.J. Biersack

*Nucleararmed University of Bonn, FRG and SBL, Stockholm, Sweden.*

This investigation was undertaken to evaluate the effect of irradiation on the binding of monoclonal anti-TPA to tumours during radioimmunodetection (RID). The monoclonal antibody 21E4 against human tissue polypeptide antigen (TPA) was radiolabelled with 125-I and injected into animals with solid HeLa cell tumours (0.2–0.3 g). For determination of uptake the animals were killed and tumours and organs removed. The activity content of the organs was calculated as g tissue$^{-1}$ uptake min$^{-1}$ to establish the specific binding and the tumour/organ ratios. Scintigraphic images were improved after irradiation and there was increased uptake of radioactivity into the tumour which might be dependent on the time delay after irradiation:

| X-ray<br>tumour weight | –<br>0.3 g | +[a]<br>0.3 g | +[b]<br>0.3 g | +[b]<br>0.2 g |
|---|---|---|---|---|
| tumour/liver | 1.7 | 5.4 | 3.8 | 4.3 |
| tumour/lung | 1.6 | 4.6 | 6.3 | 11.4 |
| tumour/kidney | 1.2 | 3.2 | 2.2 | 2.8 |
| tumour/spleen | 2.3 | 6.3 | 5.8 | 10.0 |
| tumour/ileum | 7.6 | 19.0 | 27.1 | 71.4 |
| tumour/muscle | 7.6 | 14.3 | 17.8 | 21.7 |

[a]1 day after; [b]14 days prior to antibody injection.

RID after irradiation would be of interest for patients who had undergone surgery and irradiation since remaining tumour tissues might be detected.

### CA19.9 and anti-CEA immunoscintigraphy in patients with gastric, colorectal and pancreatic malignant tumours

J.R. Garcia-Talavera, M.A. Gomez, A. Sanchez, L. Ortega, I. Rayo & J.G. Blancho

*Servicio Medicina Nuclear, Hospital Clinico Universitario, Salamanca, Spain.*

A group of 67 patients with gastro-intestinal tumours was studied: (a) 30 had gastric cancer, (b) 27 had colorectal cancer, (c) 5 had biliary or pancreatic cancer and (d) 5 had cancer of other origins. Eighty-six sites were affected: 45 primary, 18 loco-regional recurrences and 32 metastatic. $F(ab')_2$ fragments were used. Immunoscintigraphy was performed 5 days after administration of 131-I labelled antibody. Scintigraphy with $^{99m}$Tc-radiocolloid and sometimes with $^{99m}$Tc-HDP or $^{99m}$Tc-O was carried out for anatomical reference. Positive results were obtained in 25 patients in group (a), 20 in group (b), 5 in group (c) and 4 in group (d). Immunoscintigraphy was positive in 65 of 86 sites representing a 75% overall sensitivity. Immunoscintigraphy was positive in 87% of cases with elevated serum tumour markers. It was also positive in 56% of patients with negative serum tumour markers. The correlation between immunoscintigraphy and serum tumour markers was 52% for CEA and 61% for CA19.9. Three patients with no evidence of tumour; 4 with benign pathology and 2 with family history of gastric carcinoma were studied. Out of 9 patients 1 had a positive immunoscintigraphy scan. We conclude that CA19.9 and anti-CEA immunoscintigraphy was useful in the detection of colorectal, pancreatic and gastric carcinomas. This usefulness is not restricted to patients with elevated serum tumour markers and can be of interest also in patients with negative serum tumour markers.

### Potentiation of anti-CEA immunotoxin by monoclonal antibodies recognising different CEA epitopes

V.S. Byers, I. Pawluczyk, N. Berry, R.A. Robins & R.W. Baldwin

*Cancer Research Campaign Laboratories, University of Nottingham, UK and Xoma Corporation, Berkeley, CA 94710, USA.*

Immunotoxins containing ricin A chain (RTA) conjugated to anti-CEA monoclonal antibodies are cytotoxic for tumour cells expressing CEA and inhibit growth of human tumour xenografts. Immunotoxin mediated cell cytotoxicity involves multiple steps including target cell binding through the antibody moiety and endocytosis of the bound conjugate. The studies to be presented demonstrate that the cytotoxicity was determined by *in vitro* assays with gastric carcinoma MKN45 cells of an anti-CEA monoclonal antibody linked to RTA is potentiated several-fold by antibodies recognising separate epitopes on the CEA molecule. Flow cytometry analysis of the binding of fluorescein iso-thiocyanate (FITC) labelled anti-CEA antibody to gastric carcinoma MKN45 cells demonstrated that the dual treatment with second antibody produced enhanced immunotoxin binding. Furthermore endocytosis of the anti-CEA antibody labelled with tetramethyl rhodamine iso-thiocyanate (TRITC) on gastric carcinoma cells was potentiated by the dual antibody. The flow cytometry data suggest that the enhanced cytotoxicity of RTA-immunotoxin by anti-CEA antibodies recognising different epitopes reflects increased affinity of the antibody moiety of the immunotoxins. This results in prolonged retention of the immunotoxin on the tumour cells and so increases product available for endocytosis. This leads to increased cytotoxicity.

### Monoclonal antibody 791T/36-ricin A chain immunotoxin in the treatment of colorectal cancer

V.S. Byers, R. Rodvien, K. Grant & R.W. Baldwin

*Xoma Corporation, Berkeley, CA 94710; Pacific Medical Center, San Francisco, CA, USA and Cancer Research Campaign Laboratory, University of Nottingham, UK.*

Monoclonal antibody 791T/36 recognises a gp72 antigen expressed upon colorectal carcinoma cells and has been used extensively for imaging primary and metastatic colorectal cancers. This has led to the design of an immunotoxin containing ricin A chain (RTA) linked to the antibody via a disulphide bond. The immunotoxin retains a high level of antibody reactivity as determined by a competitive flow cytometry assay. This compares the ability of immunotoxins and free antibody to inhibit binding of fluorescein isothiocyanate-labelled 791T/36 antibody with 791T tumour cells. Colony inhibition znd 75Se-selenomethionine-incorporation assays demonstrate that the immunotoxin is cytotoxic *in vitro* for tumour cells expressing the gp72 antigen. The therapeutic efficacy of the immunotoxin has been demonstrated by showing that it suppresses growth of human tumour xenografts in athymic mice. Based upon these findings and related animal toxicology studies, a phase I clinical trial in patients with colorectal cancer has been carried out.

### Tumour associated antigens (TAA) and immunoscintigraphy (I-131-Anti-CA19-9) during treatment of xenografts of pancreatic carcinomas (PACA)

R. Klapdor, M. Bahlo & J.S. Kuhl

*Ch. Ufer Medical Department, University of Hamburg, FRG.*

We have studied the secretion of TAA into the serum and their cellular expression in 3 different human PACA xenografts during treatment with various modalities [chemotherapy, monoclonal antibodies (MAB), tumour necrosis factor (TNF) and interferons (IFN)].

Treatment was for 3 weeks with: Mitomycin-C i.p., Mab bw 494/32 or 17-1A 400 $\mu$g day$^{-1}$ and 300,000 U day$^{-1}$ given i.p. For immunoscintigraphy (tumours of ISCH 84) we injected 100 $\mu$Ci I-131-anti-CEA 19-9 into the tail vein. The results demonstrate that in the nude mouse model concomitant therapy may result in a significant decrease of TAA in the serum correlating with decreasing tumour size in some cases but also with tumour progression in other cases (HJ 84). This decrease of serum TAA does not necessarily represent evidence of tumour regression. It was of interest that different treatment modalities had a different effect on circulating TAA (e.g. TNF induced a more marked decrease of serum CA19-9 than mitomycin-C).

### Immunotherapy of pancreatic carcinomas (PACA) with the monoclonal antibody BW494/32

R. Klapdor, M. Bahlo, O. Schwarzenbeck & M. Hirschmann

*Medical Department, University of Hamburg, FRG.*

BW 494/32 is a monoclonal antibody which might allow treatment of PACA because of (a) positive ADCC in Cr-51-release assay, (b) positive antigen expression by highly or moderately differentiated PACA, (c) positive immunoscintigraphy with I-131-BW494/32 in patients as well as in xenografts in nude mice and (d) dose dependent inhibition of tumour growth of xenografts. We studied 10 patients suffering from advanced PACA: 8 male, 2 female, 44–73

years of age. Six had prior chemo- or radiation therapy. Five patients were treated with increasing doses up to a total of 150–210 mg, and 5 with doses up to 500 mg (30 mg day$^{-1}$ after 100 mg at day 1). Mab levels in serum were measured during and after treatment as well as human anti-mouse response (HAMA). Two patients had allergic reactions (1 after the 4th application, 1 after the 3rd cycle) but therapy in general was well tolerated. Mab serum concentrations were 20–40 $\mu$g ml$^{-1}$. All patients studied so far developed HAMA. Two patients showed a significant decrease of CEA and in 1 case there was regression of liver metastasis and primary tumour necrosis.

### Immunotherapy of pancreatic cancer with the monoclonal antibody BW 494/32 – preliminary results of a phase I/II clinical trial

R. Kubel, M. Buchler, G. Schulz, K. Bosslet & H.G. Beger

*Dep. General Surgery, University Ulm, and Behring Werke AG, Marburg, FRG.*

The hybridoma derived murine monoclonal antibody BW 494/32 was applied in a phase I/II clinical trial in passive immunotherapy in patients with un-resectable pancreatic cancer. This antibody was selected because of its ADCC (antibody-dependent-cellular-cytotoxicity) reactivity against established pancreatic cancer cells *in vitro* and its high sensitivity and specificity for human pancreatic carcinoma tissues as demonstrated by immunohistochemistry and immunoscintigraphy. Between 5/86 and 2/87, 14 patients with proven unresectable pancreatic cancer entered the study. Postoperatively they received various dosages of antibody i.v. – either in increasing dosages up to 300 mg in 5 days (group I) or as a constant dosage at 3 day intervals up to 300 mg within 3 weeks (group II). All treated patients were included in a follow-up program. Second-look operations were performed in 3 cases. During primary antibody therapy, 2 patients in group II required treatment for severe allergic reactions, probably due to a human anti-mouse response (HAMA), starting ~2 weeks after the first antibody application. Elevated HAMA levels could be measured for months.

Out of 11 evaluable patients, 3 had a favourable clinical course with evidence of 'stable disease'. One patient had no evidence of tumour progression assessment second-look operation 8 months after antibody administration. Eight patients with high tumour load did not respond.

Criteria for the selection of patients with pancreatic cancer who are suitable for immunotherapy have to be established. HAMA response limits repeated antibody administration.

### Preliminary biodistribution studies of monoclonal antibody immunoconjugates

A.C. Perkins, M.V. Pimm, K.C. Ballantyne, J.D. Hardcastle & R.W. Baldwin

*Departments of Medical Physics, Surgery and Cancer Research, University Hospital, Nottingham, UK.*

The use of monoclonal antibody-drug conjugates to selectively increase the delivery of drug to tumours whilst reducing whole body toxicity is currently being assessed. Targetting studies are currently underway to assess the tumour localisation and whole body biodistribution of the anti-P72,000 dalton glycoprotein monoclonal antibody 791T/36 conjugated to cytotoxic drugs including methotrexate (MTX) and a plant-derived toxin.

Preliminary studies in mice with human tumour xenografts have demonstrated the preferential localisation of radiolabelled immunoconjugates. Tumour localisation of the immunotoxin preparations has not been visualised and accumulation of the toxin by the liver appears to be the main problem although a therapeutic effect has been observed in the mouse xenograft model.

Clinical imaging studies in 15 patients with colorectal cancer injected with 70 MBq I-131-labelled 791T/36-methotrexate (200 $\mu$g antibody:1.6 $\mu$g MTX) have demonstrated that the biodistribution of the drug-conjugate is similar to that of the unconjugated antibody. A T:NT uptake ratio of 2.9:1 has been measured from resected specimens. However, an assay is being developed to determine the concentration of drug in tumour and normal tissues.

Further clinical studies will be necessary to assess the targetting ability of antibody-drug conjugates prior to their use for tumour therapy.

### Therapeutic strategies with biologically targetted radiotherapy

J.A. O'Donoghue & T.E. Wheldon

*GIRO, Belvidere Hospital, Glasgow, UK.*

Biologically targetted radiotherapy (BTR) with radiolabelled antibodies or biochemical molecules such as MIBG has the potential to improve the treatment of neoplastic disease. The area where there is the greatest theoretical promise is in the treatment of systemic disease where surgery or local radiotherapy are not applicable. In common with other forms of radiation therapy, the factor determining allowable radionuclide doses will be the capacity of normal organs to withstand radiation insult. Current experience with BTR indicates that bone marrow is the dose-limiting organ. By analogy with external beam total body irradiation (TBI) it is expected that doses could be substantially increased by incorporating bone marrow rescue (BMR) into treatment protocols. Using the radionuclide 131-I it is anticipated that a therapeutically advantageous biological heterogeneity would be imposed by replacing one or more fractions of a multifractionated TBI schedule by an appropriate amount of BTR. Future development of BTR will probably involve the use of alternative radioisotopes such as 90-Y and 211-At. Mathematical model studies indicate that combination TBI/BTR schedules incorporating BMR may be the optimal strategy for treatment of systemic malignancy using these radioisotopes.

### Theoretical study of the possibility of biologically targetted hyperthermia by hysteresis heating of antibody-linked magnetic particles

J.A. O'Donoghue, T.E. Wheldon, H. Moseley & G. M. Ford

*GIRO, Belvidere Hospital, Glasgow, UK.*

The possibility of using metallic implants or injections of ferromagnetic particles as a focus of heat generation by oscillating magnetic fields of RF energy has been known for some time. Difficulties associated with the injection of material include possible toxicity of the material, non-uniformity of dosing and the tendency for the materials used to remain at the injection site. No biological specificity is involved in any of the methods reported to date. A new type of 'warhead' for antibody-guided cancer therapy may allow biologically specific delivery of hyperthermia to tumour cells. An example of this type of warhead is provided by the monodisperse polymer particles incorporating a quantity of magnetite which are presently used in bone-marrow purging procedures. This approach has the theoretical advantage that external 'activation' of the warhead is required which may allow sparing of normal organs (e.g. liver). Simple mathematical model studies suggest that this concept is not implausible in some circumstances. Significant heating of very small conglomerations and particularly individual cells is unlikely. The most suitable targets appear to be conglomerations of tumour cells of millimetre dimensions. Though undeniably speculative, biological targetting of hyperthermia seems worthy of further study using more realistic mathematical models and experimental investigation *in vitro*.

### Immunotargetting in infective *vis-à-vis* neoplastic lesions

D.K. Hazra, V. Lahiri, B.V.R. Elhence, K. Singh, R.N.L. Srivanstava, I.P. Elhence, S. Saran, J.P.K. Bhattacharjee, B. Arvind, S. Khandelwal, M. Kumari, V.K. Rohtagi & R. Singh

*S.N. Medical College, Agra, India.*

Radioimmunotargetting in neoplastic lesions using monoclonal antibodies is closely paralleled by the use of such antibodies in targetting infective lesions. The relative problems in using these two models were studied using 125-I labelled antimycobacterial tuberculosis antibodies WTB72 and WML34 and HMFG2 antibody in experimental models (tuberculomas in normal Swiss albino mice and xenografted human tumours in immunosuppressed mice). In infective lesions the value of radioimmunotargetting is largely diagnostic whereas for neoplasms both imaging as well as therapy have to be considered. The latter involves consideration of residence times in both tumour as well as in critical organs. Successful radioimmunolocalization in tuberculous lesions is especially significant in the Indian context where tuberculomas form an important differential diagnosis of neoplastic lesions. Radioimmunotargetting in infective lesions may be easier than neoplastic lesions because tumour cells are more likely to share antigens with normal tissues.

### Radioimmunoscintigraphy (RIS) of ovarian cancer with Iodine 121 labelled $F(ab')_2$ fragments of anti-CEA and OC125

G. Barzen, M. Langer, R. Becker, A. Mayer, K. Koppenhagen & R. Felix

*Radio-Immuno-Scintigraphy Group, Free University of Berlin, FRG.*

We are using an I-131 labelled cocktail of 1 mg anti-CEA and OC125 $F(ab')_2$ fragments with a total activity of 80–120 MBq (IMACIS 2/CIS-Isotope Diagnostic).

We investigated 30 patients after primary treatment of ovarian cancer and 8 women preoperatively. Scanning was done 1–7 days after application of the cocktail with SPECT and planar technique. RIS was proven by CAT-Scan and surgical means. We calculated the tumour to nontumour tissue ratio (T/N) scintigraphy at the 6th day p.i.

In the follow-up 21 of 30 women had a relapse and/or metastasis in the true pelvis. Eighteen had a true positive scan. The T/N ratio was 1.6 and 2.2 (mean 1.8). Two patients had a false positive scan, one proven pathologically (Figo stage 3, RIS and biopsy after second look and chemotherapy). Three scans were false negative, T/N ratio 1.3; 1.3; 1.4.

In 16 patients metastatic disease was found outside the true pelvis. In 13 we found a true positive scan. The T/N ratio was between 1.8 and 2.8 (mean 2.0). Three scans were false negative (lung/liver/liver). Eight scans were false positive: increased liver uptake (3), cyst in the liver (1), ascites (1), increased paraaortic uptake (lymphnodes) (3) CAT-scan: no disease.

RIS is a useful diagnostic tool in the follow-up management of ovarian cancer; nevertheless the T/N ratio is low.

### The importance of low plasma CEA values in healthy smokers for the sensitivity of CEA-assays in patients with cancer

P. Oehr

*Inst. Nuclearmed, D-5300 Bonn, FRG.*

The use of panels of monoclonal antibodies for plasma CEA immunoassays enables further improvement of epitope specificity. As a result, an increased diagnostic sensitivity might be achieved compared to immunoassays using polyclonal antibodies. Three poly and 8 monoclonal CEA immunoassays were used simultaneously to determine the plasma levels in healthy individuals ($n = 149$; 74 smokers and 75 non-smokers) and patients with cancer ($n = 92$). The results as per assay were transformed into inverse distribution curves and receiver operator characteristic (ROC) curves to determine sensitivity and specificity of the assays. One of the monoclonal CEA tests showed a superior sensitivity compared to all the other tests. Further analysis of the data revealed that the improvement of discrimination was not due to increased sensitivity but to better specificity in the control group. The antibodies applied did not lead to as many elevated CEA values for smokers as those of the other tests. This results in a 100% increase of sensitivity compared to the other CEA test kits. This shows that exclusion of 'false positive' CEA values in the control group is important for increasing the discrimination of CEA-assays.

### Immunocytochemical detection of tumour cells in the bone marrow of breast cancer patients at the time of primary therapy

N. Harbeck, M. Untch & W. Eiermann

*Frauenklinik im Klinikum Grobhadern der LMU Munchen, 8000 Munchen 70, FDR.*

Bone marrow aspirates from 45 patients and trephine biopsies from 18 patients were taken at the time of primary breast cancer therapy (stage T1–3, NO–2, MO–1). All patients were screened for distant metastasis (X-ray, bonescan).

Tumour cells in the bone marrow aspirates were detected by immunohistochemistry using monoclonal antibodies against EMA (epithelial membrane antigen) and LICR-LON-M8 (kindly provided by the Ludwig-Institute, Sutton, UK).

In 15/45 patients, bone marrow aspirate staining of tumour cells (6–32 cells) could be demonstrated. Four of the 15 patients were at stage I, 9 at stage II, one at stage III and one at stage IV disease at the time of clinical presentation. In the 18 trephine biopsies, tumour spread could not be detected. The median follow-up time was 13.1 months (min. 3 months, max. 27).

Eight of the 15 EMA-positive patients developed bone metastases and also had visceral involvement. Two out of the 30 EMA-negative patients developed local recurrence, one visceral metastasis, and one bone metastasis. The median time from bone marrow sampling to metastasis in EMA-positive patients was 6.5 months. To detect the overall survival rate longer follow-up periods and greater patient numbers are required.

We conclude that bone marrow sampling can detect bone micrometastasis at the time of primary therapy.

### The nude rat heterotransplanted with human malignant melanoma – an experimental model evaluating factors influencing the kinetics of radiolabelled monoclonal antibodies

S.E. Strand, C. Ingvar & Norrgren

*Radiation Physics Department, Surgery Department, University of Lund, Lund, Sweden.*

The nude rat, being larger, less sensitive and longer lived than the nude mouse, offers advantages for studies of tumours with radiolabelled monoclonal antibodies.

Nude rats (Rowett RNu/RNu) 2–3 months of age, weighing 200–250 g were used. A human melanoma metastasis (UM) maintaining strong expression of p97 melanoma-associated cell surface antigen was used. The tumour was inoculated both s.c. and i.m. Monoclonal antibodies 96.5, 2B2 and MG-21 as whole antibodies and fragments and control antibody 1.4 have been used. The antibodies were injected either i.v. or s.c. To assess the blood content in the tissues and to correct for circulating antibodies red blood cells were labelled with 99-Tc-m. Scintillation camera imaging was performed over one week and animals were regularly dissected for specific organ retention studies. This model has proven to be easier and more useful to work with than the nude mouse model.

### Antibody guided targetting of non small cell lung cancer using Indium-111 HMFG1-F(ab′)$_2$ fragments

H.P. Kalofonos, G. Sivolapenko, N. Courtenay-Luck, D. Snook, G. Hooker, R. Winter, C.G. McKenzie, J. Taylor-Papadimitriou, J.P. Lavender & A.A. Epenetos

*ICRF Oncology Group, Department of Clinical Oncology, Respiratory Unit, Medical Physics, Diagnostic Radiology, Hammersmith Hospital and Imperial Cancer Research Fund, London, UK.*

Immunoscintigraphy using F(ab′)$_2$ fragments of tumour associated monoclonal antibody HMFG1 was performed in 14 patients with primary and metastatix non-small cell (NSCC) lung cancer. The antibody was conjugated with DTPA and labelled with Indium-111.

All patients had significant concentration of Indium-111 in the liver. No toxicity was encountered. No human anti-murine-IgG antibody was detected in patients receiving 2 administrations of F(ab′)$_2$ fragments.

Localisation of all primary lesions and the majority (80%) of metastatic lesions was achieved. Seven out of 14 patients were also studied using an 111-In-labelled non-specific antibody F(ab′)$_2$ fragments. In 3 patients, the uptake of the non-specific antibody was significantly lower than that of specific antibody ($P<0.05$). In the other 4 patients there was no significant difference in the uptake between specific and non-specific antibodies.

We conclude that although successful targetting of 111-In-labelled F(ab′)$_2$ fragments of HMFG1 can be achieved in patients with NSCC of lung, significant tumour localisation can also be achieved using a non-specific antibody.

### The use of CA-50 radioimmunoassay inhibition test in the differential diagnosis of benign and malignant disease of the breast, stomach and oesophagus

S.B. Kelly, M.J. Hersham, N.A. Habib, R.C.N. Williamson, J. Spencer & C.B. Wood

*University Department of Surgery, Bristol Royal Infirmary, Bristol, and Department of Surgery, Royal Postgraduate Medical School, London, UK.*

This study investigated the role of the tumour marker CA-50 (carcinoma associated antigen) in the differential diagnosis of benign and malignant disease of the breast, stomach and oesophagus. Serum was collected from 50 controls, 24 patients with benign breast disease, 19 with benign oesophago-gastric disease, 26 with breast carcinoma, 24 with breast carcinoma, 24 with gastric carcinoma, and 21 with oesophageal carcinoma. A radioimmunoassay (RIA) was used to detect CA-50 in the serum and a level of 17 U ml$^{-1}$ was used as a cut-off between benign and malignant disease. All 50 normal subjects, 22 of 24 patients (92%) with benign breast disease and 18 out of 19 (95%) with benign oesophago-gastric disease had CA-50 levels below 17 U ml$^{-1}$. In the cancer groups, 15 of 36 (42%) with breast carcinoma, 18 of 24 (75%) with gastric carcinoma and 15 of 21 (71%) with oesophageal carcinoma had CA-50 levels above 17 U ml$^{-1}$. Therefore in the breast group, the sensitivity is 42% (15/36) and the specificity is 100% (50/50) and 92% (22/24) for the control and benign groups respectively. In the stomach and oesophageal group the sensitivity is 73% (33/45) and the specificity is 100% (50/50) and 95% (18/19) for the control and benign groups respectively. In the group with breast carcinoma, there was no clear correlation with clinical stage: 6/12 (50%) with stage I disease had CA-50 levels >17 U ml$^{-1}$, 5/13 (39%) stage II, 1/4 (25%) stage III and 3/7 (43%) stage IV. The data suggest that the CA-50 RIA test could be of no use in the differential diagnosis of benign and malignant diseases of the breast, stomach and oesophagus.

### Elevated serum HMFG-levels in breast and ovarian cancer patients measured with a sandwich ELISA

P. Ashorn[1], O. Kallioniemi[2], T. Hieranen[3], R. Ashorn[4] & K. Krohn[1]

[1]*Institute of Biomedical Sciences, University of Tempere;* [2]*Department of Clinical Chemistry, Tampere University Central Hospital;* [3]*Department of Obstetrics and Gynaecology, Tampere University Central Hospital and* [4]*Department of Radiology, Tampere University Central Hospital, Finland.*

HMFG antigen is a tumour-associated glycoprotein that has been shown immunohistochemically to be expressed by malignant cells in breast and ovarian and to a lesser degree in gastrointestinal carcinomas. In this study we have developed a non-isotopic sandwich ELISA for HMFG antigen utilizing a polyclonal catcher and a monoclonal tracer antibody. 52/52 healthy medical students had a serum value <400 U ml$^{-1}$ whereas 15/30 patients (50%) with evidence of ovarian cancer and 13/37 (35%) with advanced breast cancer had a value exceeding 400 U ml$^{-1}$. 2/14 (14%) patients with uterine cancer, 0/5 with cervical cancer, 0/5 with vulva carcinoma, 1/33 with gastrointestinal cancer, 0/4 with oesophageal cancer and 2/45 patients with leukaemia or lymphoma had serum values below 400 U ml$^{-1}$. Progression of ovarian cancer correlated with elevation of HMFG antigen levels. Comparison of serum values of HMFG antigens to CA125 and CA15-3 values showed that the antigen detected by our assay is different from CA125 but may be overlapping with CA15-3 antigens.


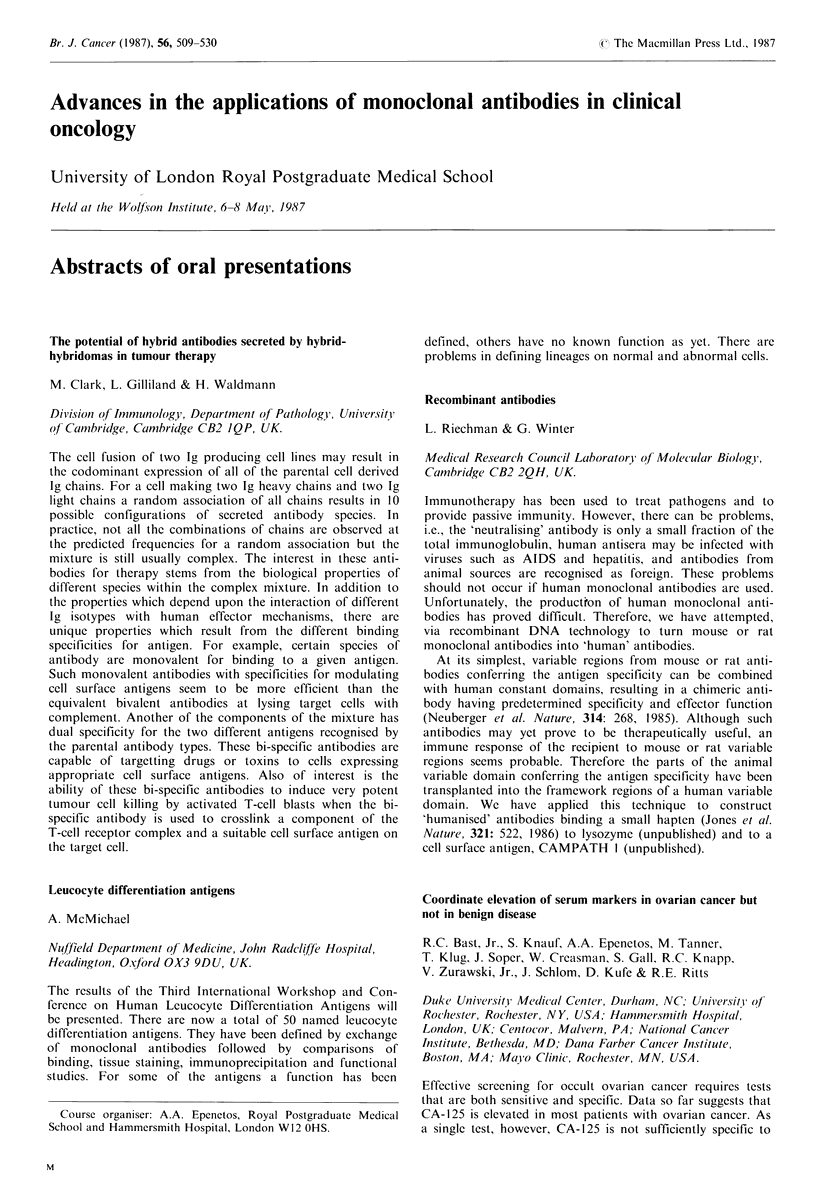

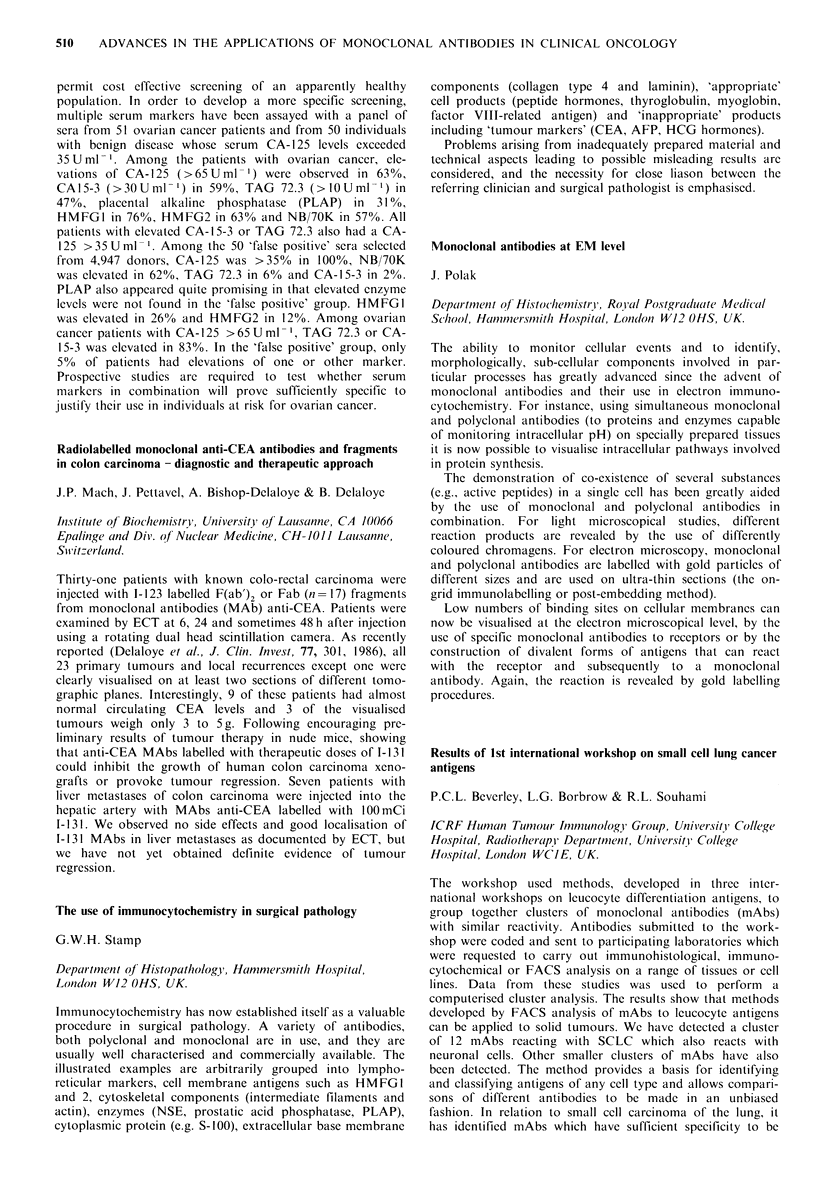

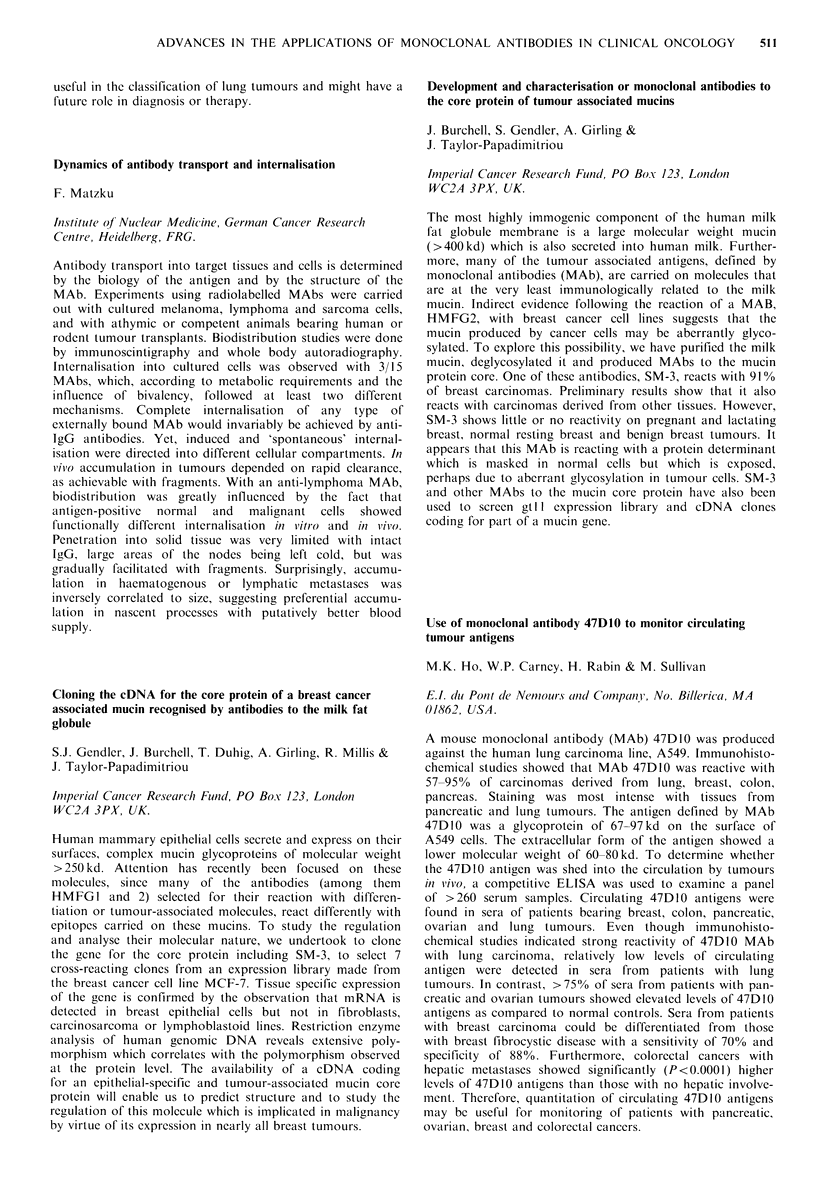

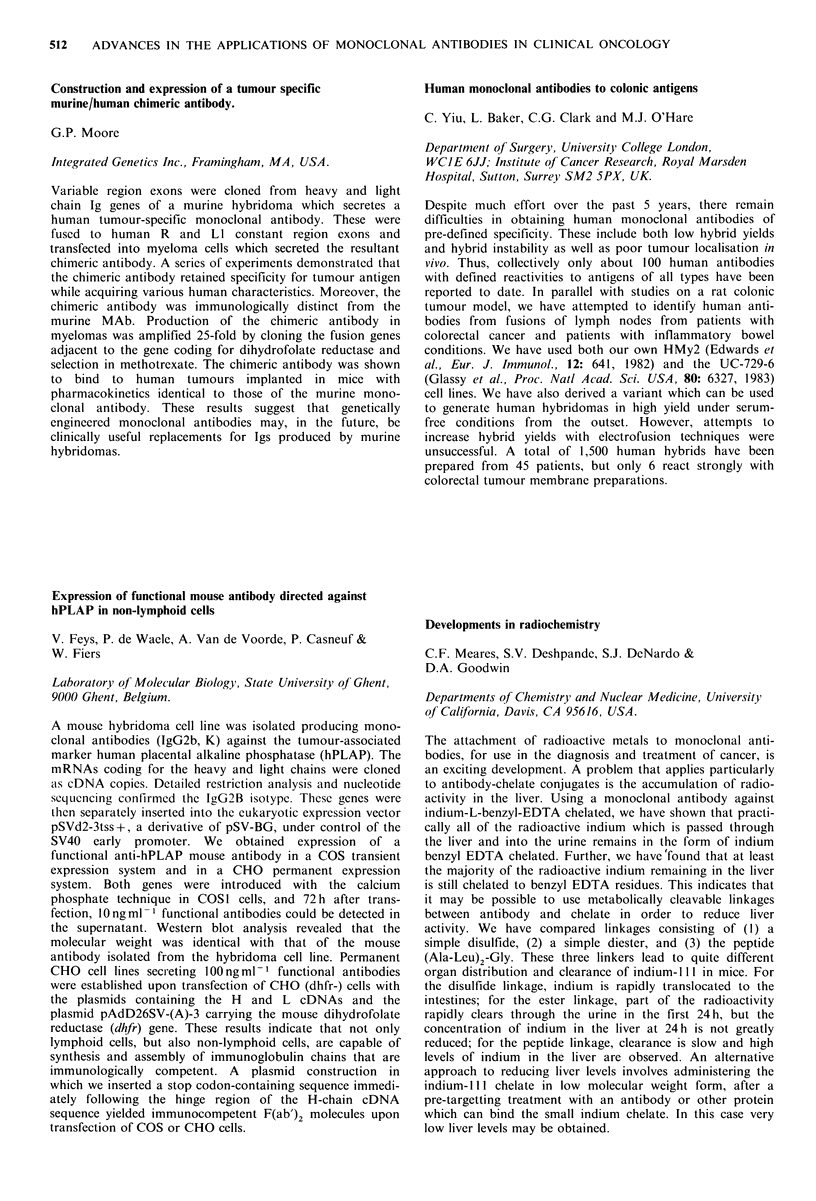

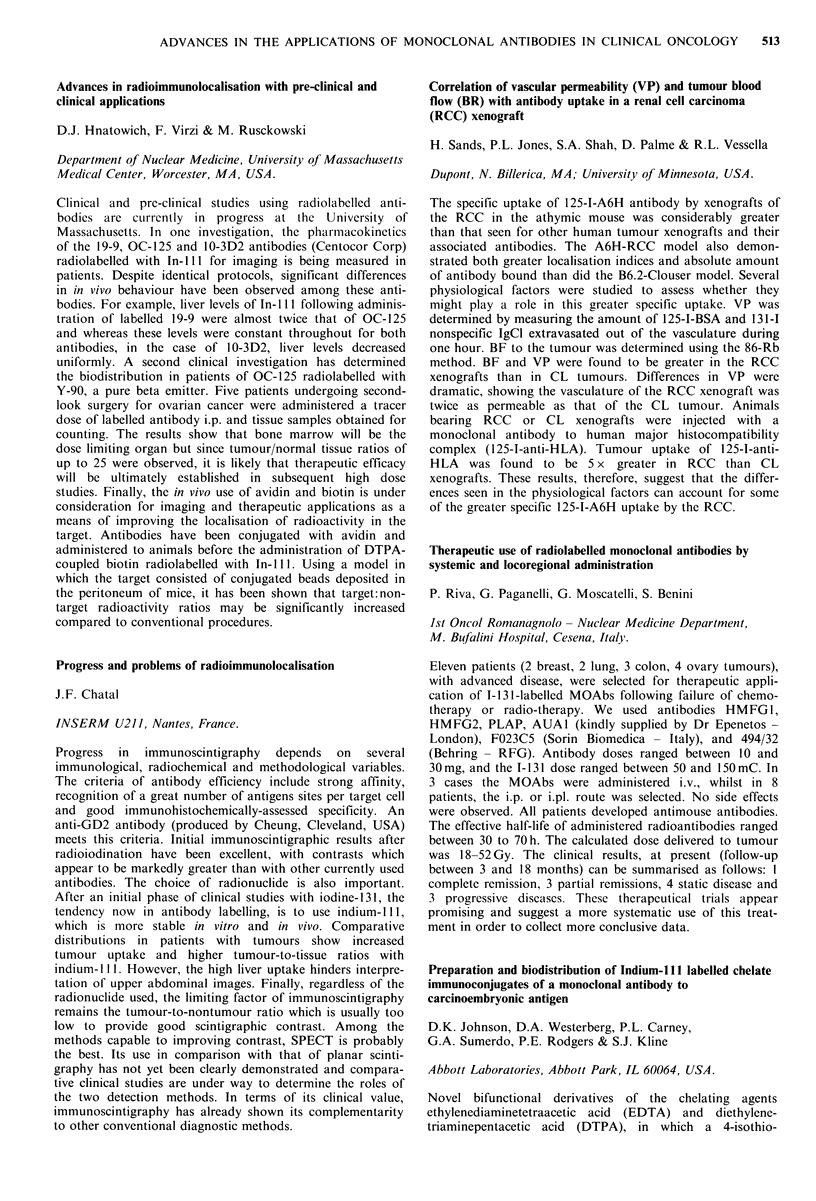

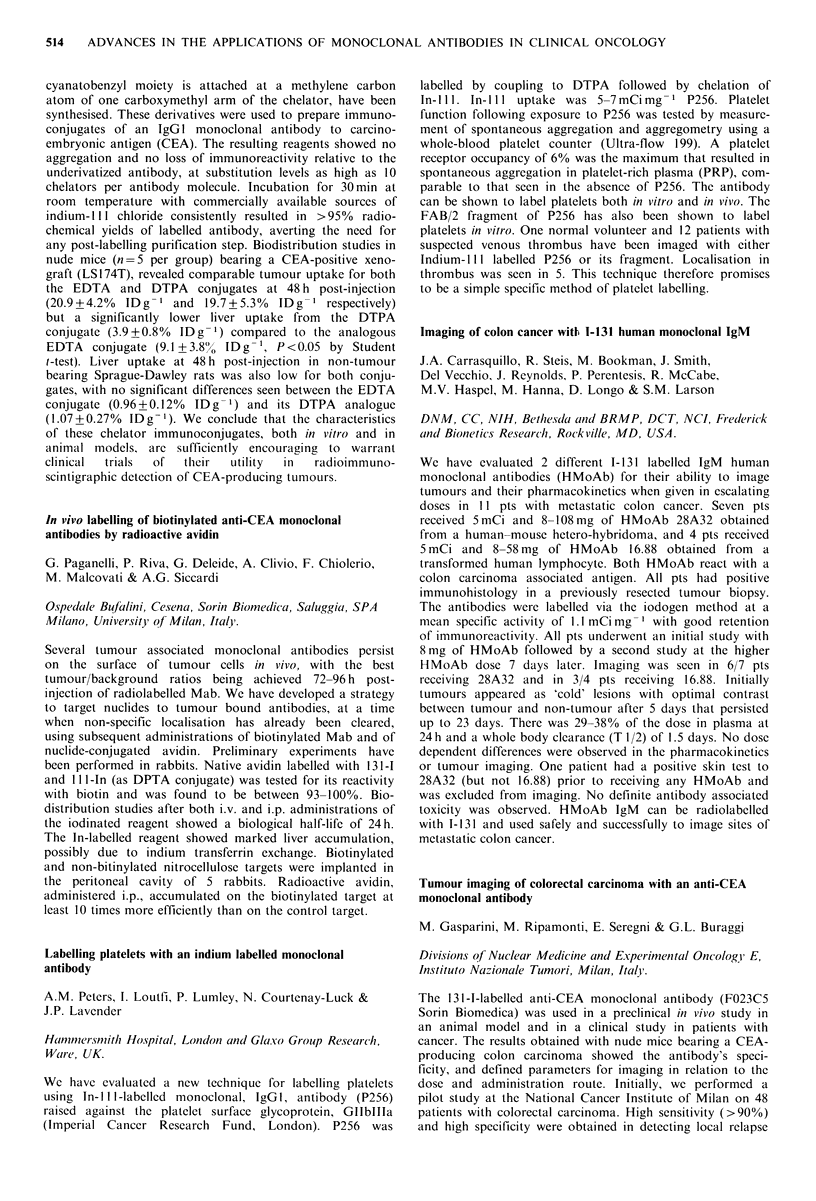

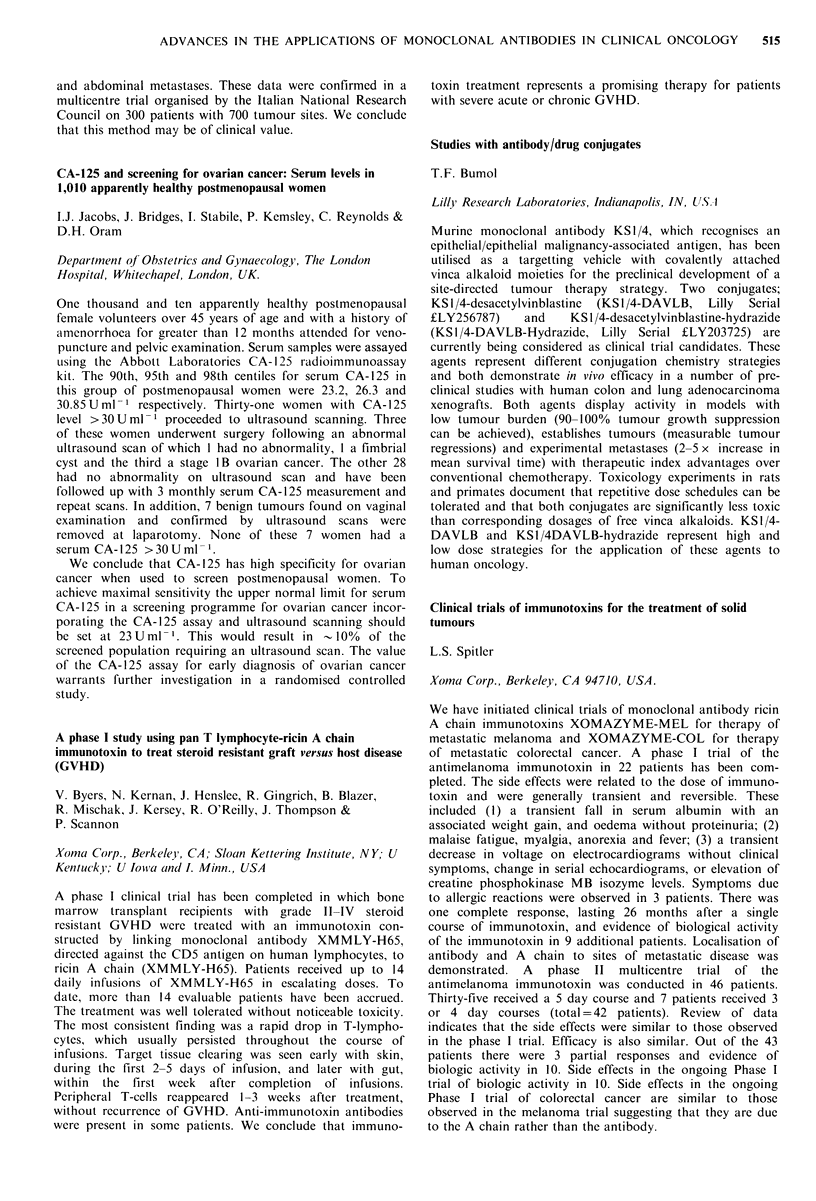

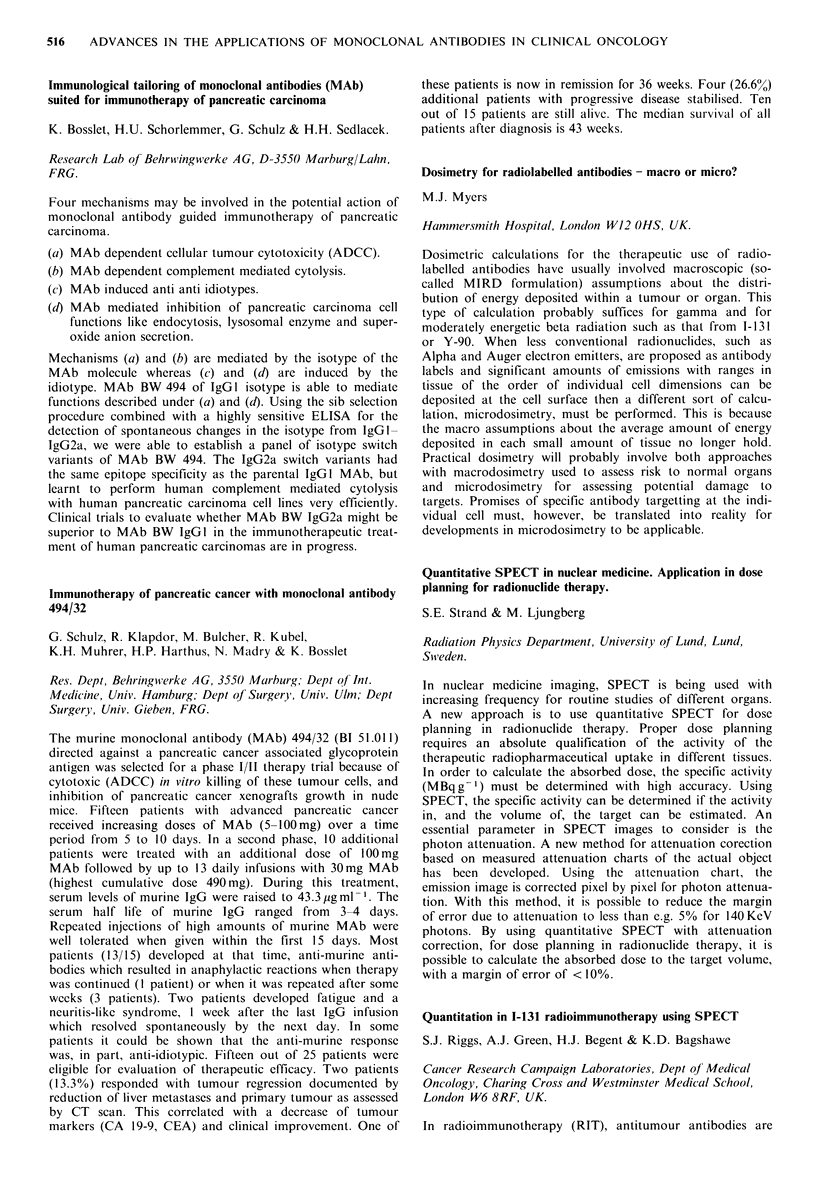

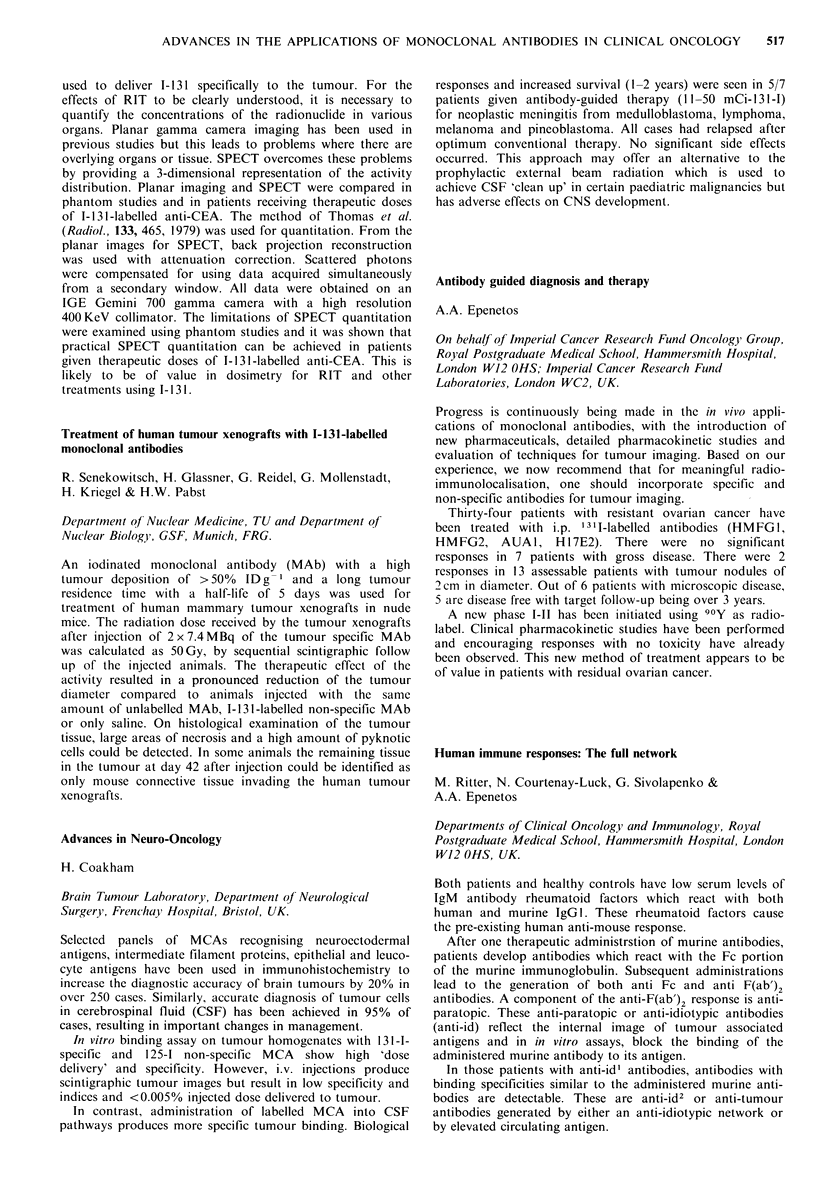

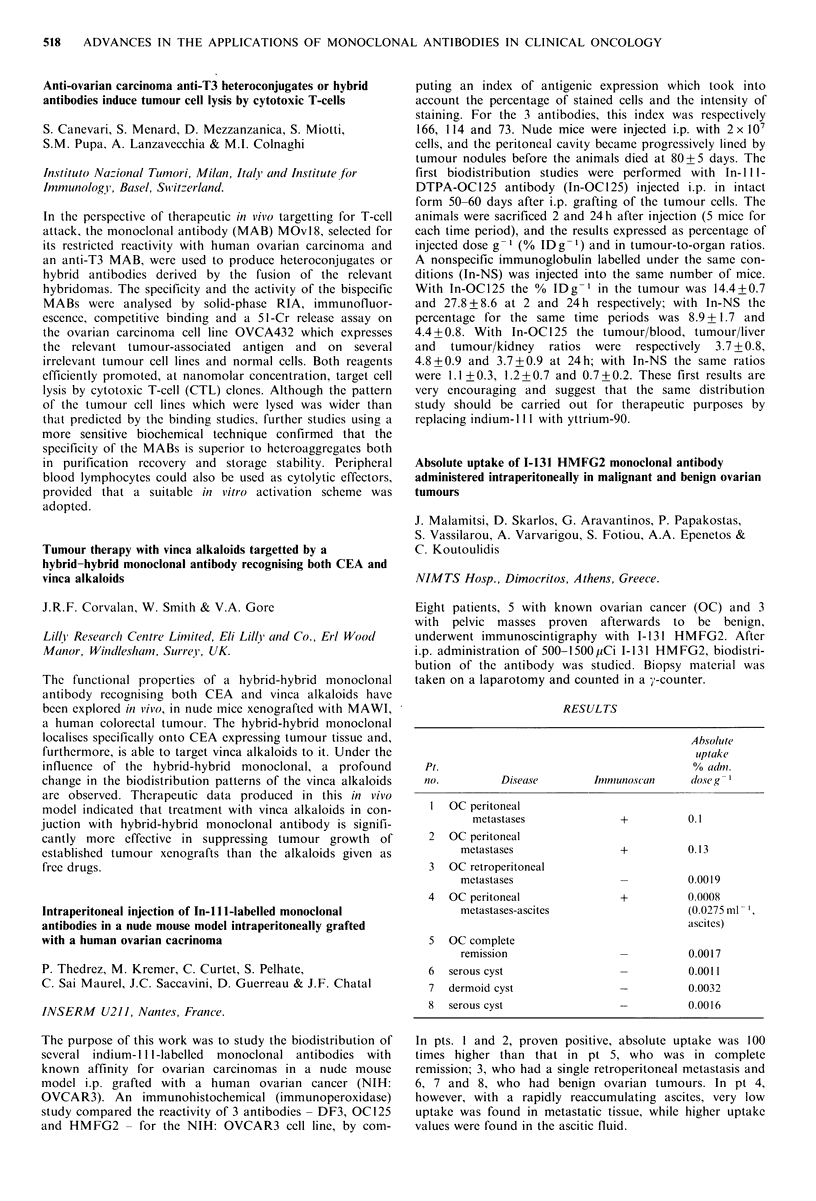

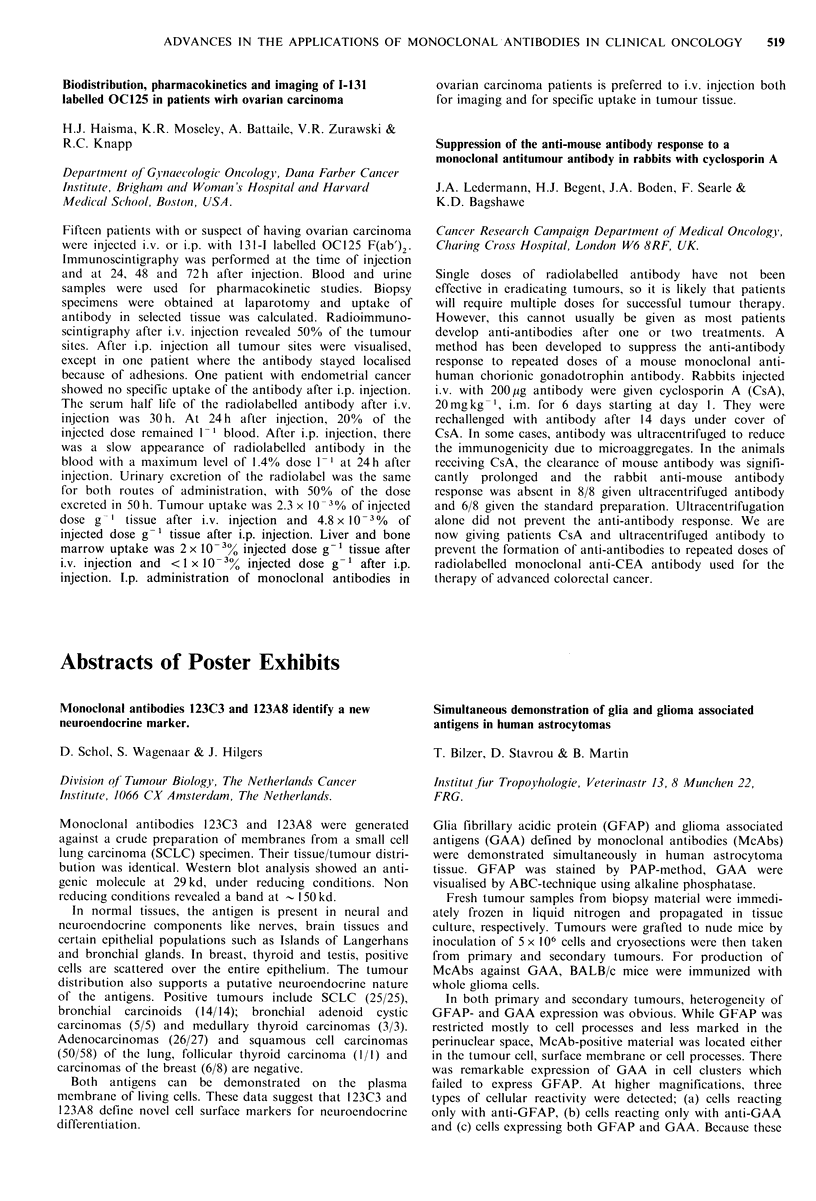

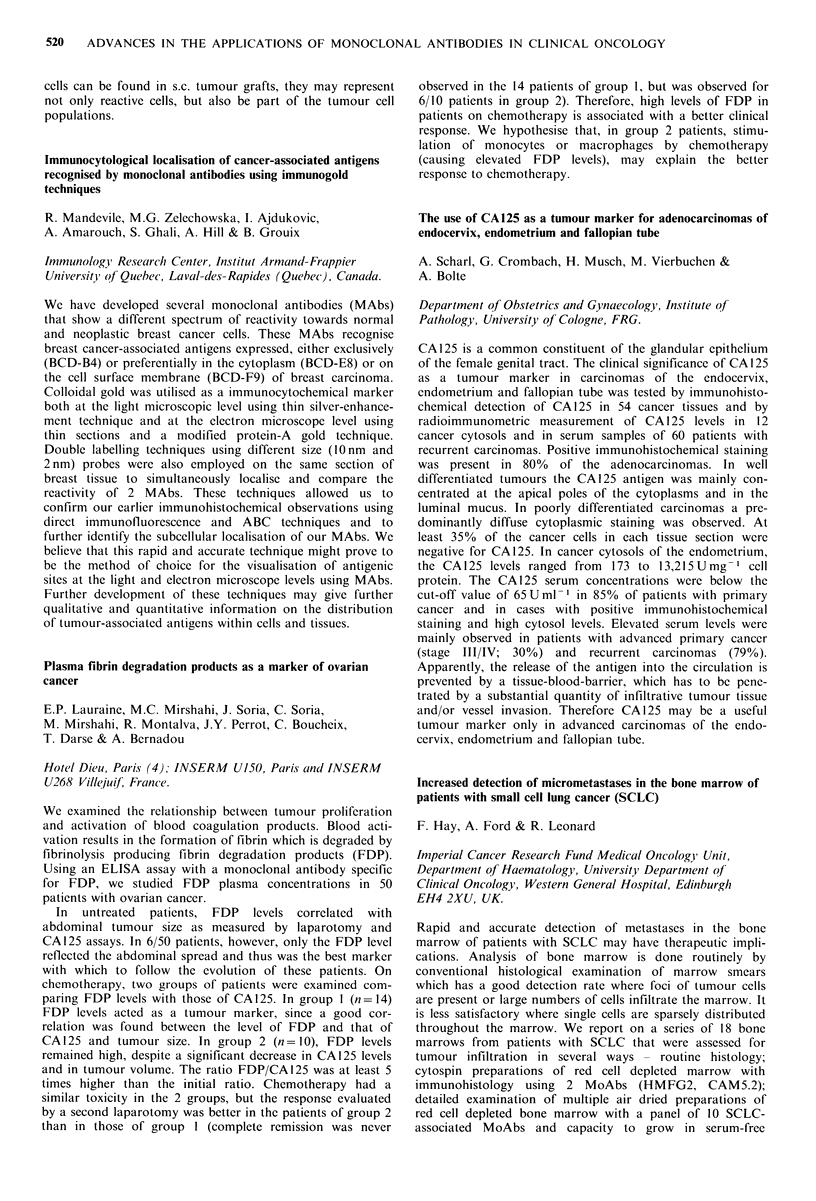

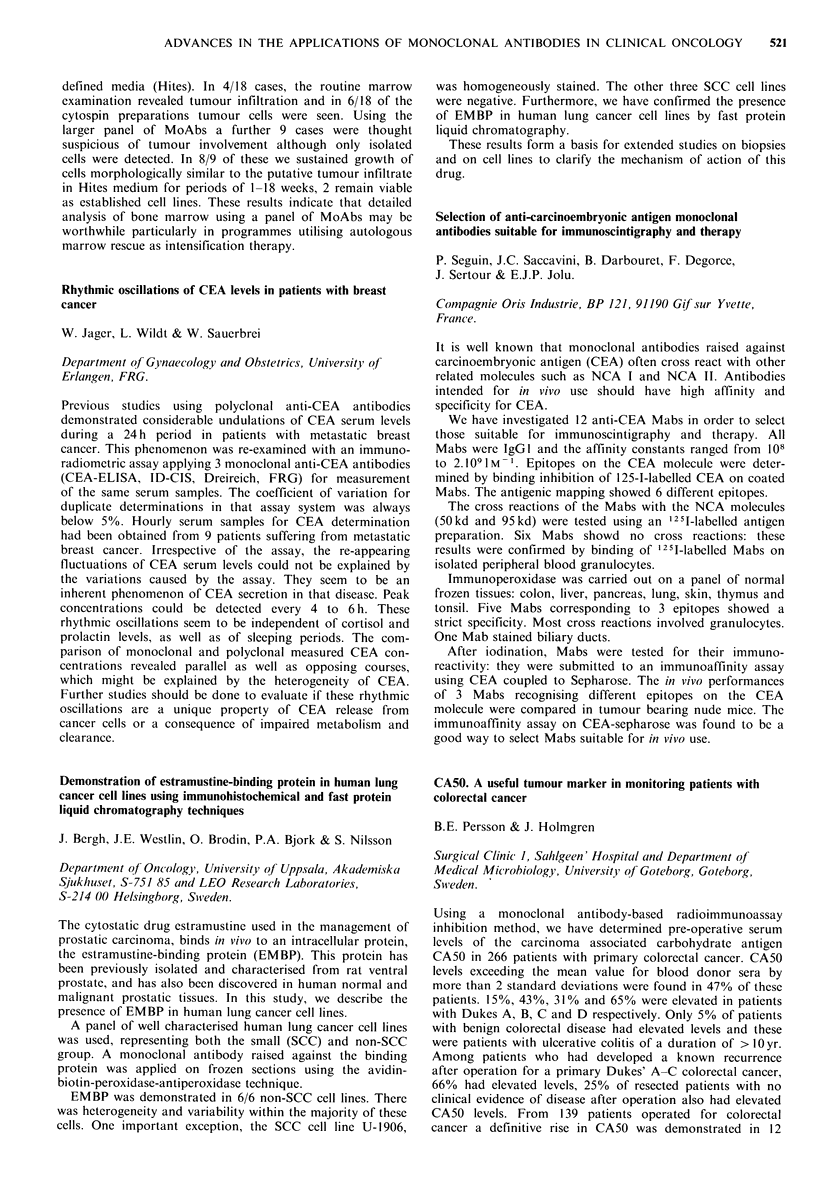

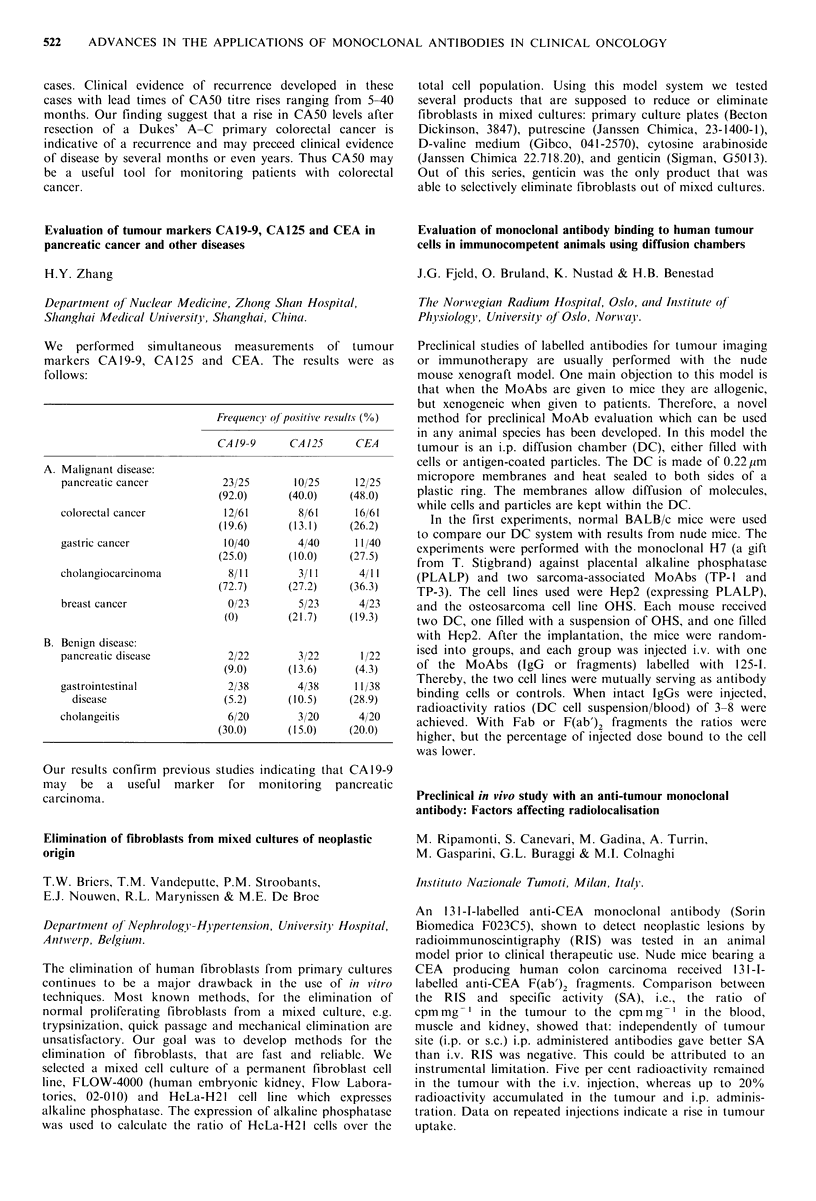

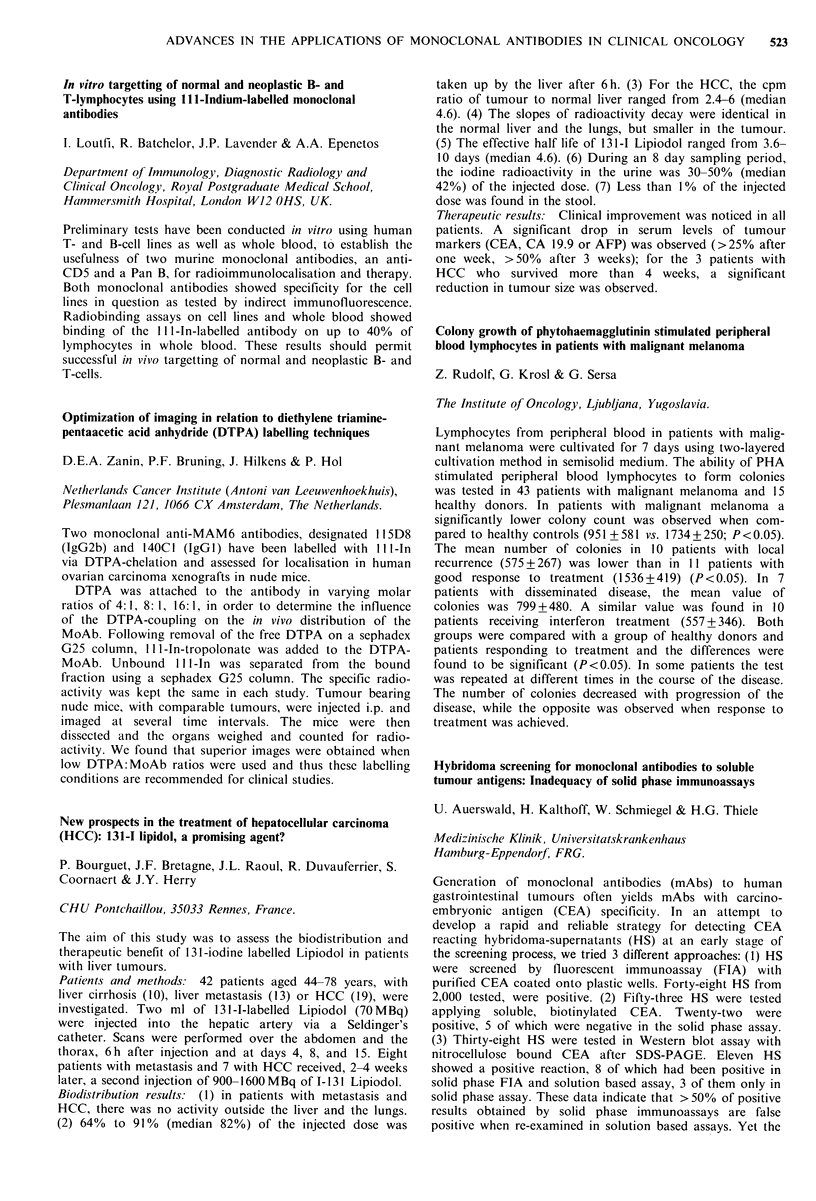

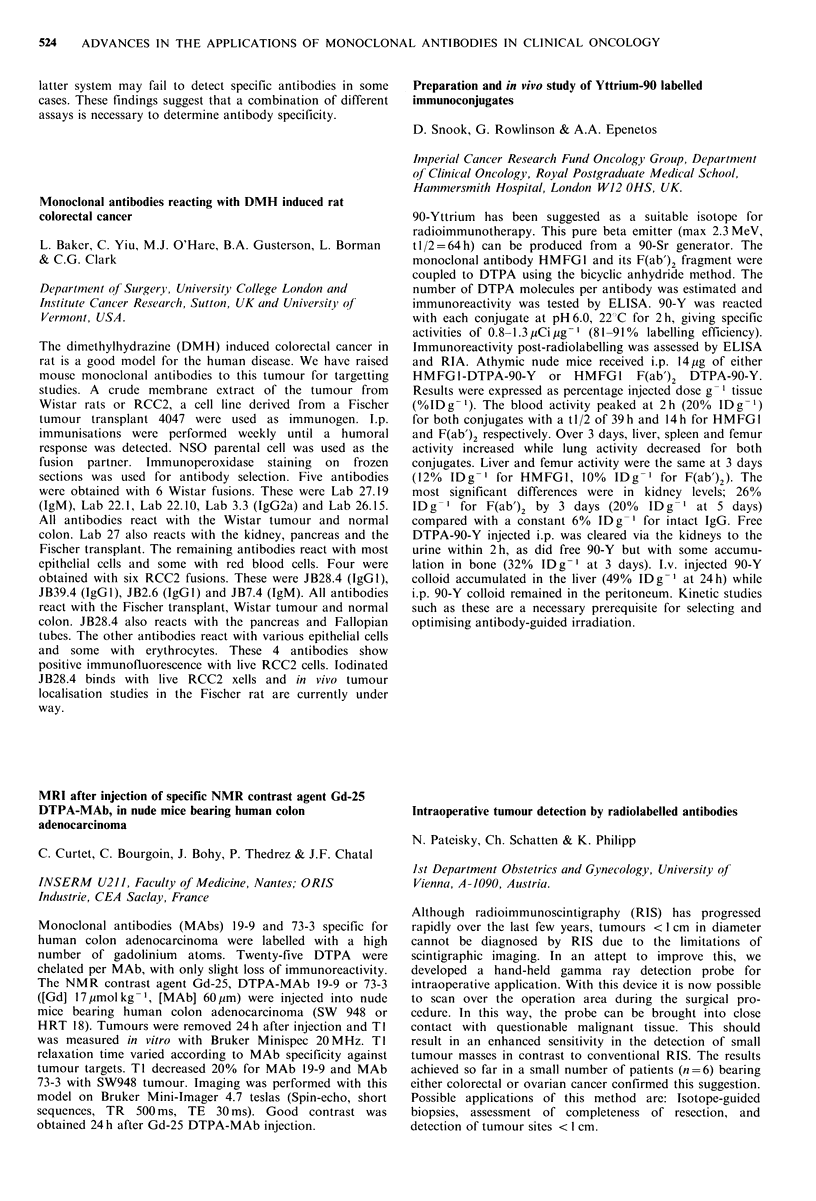

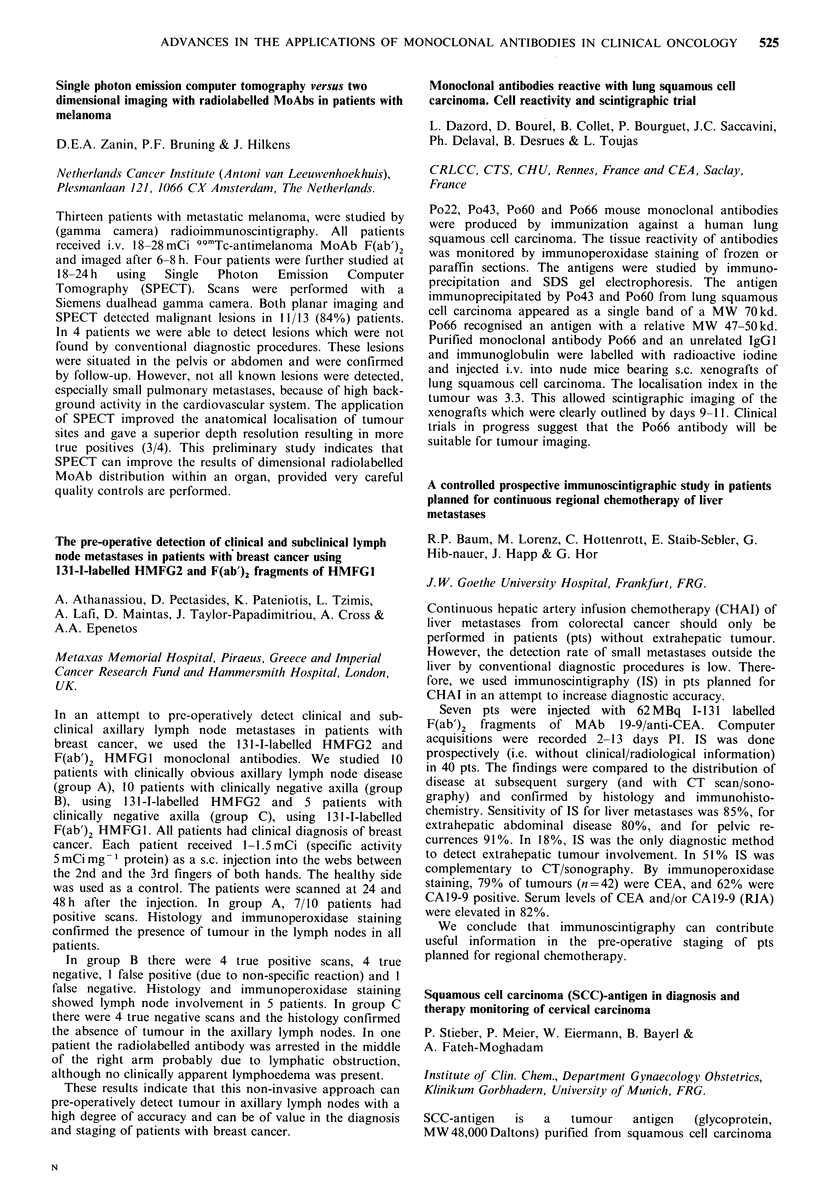

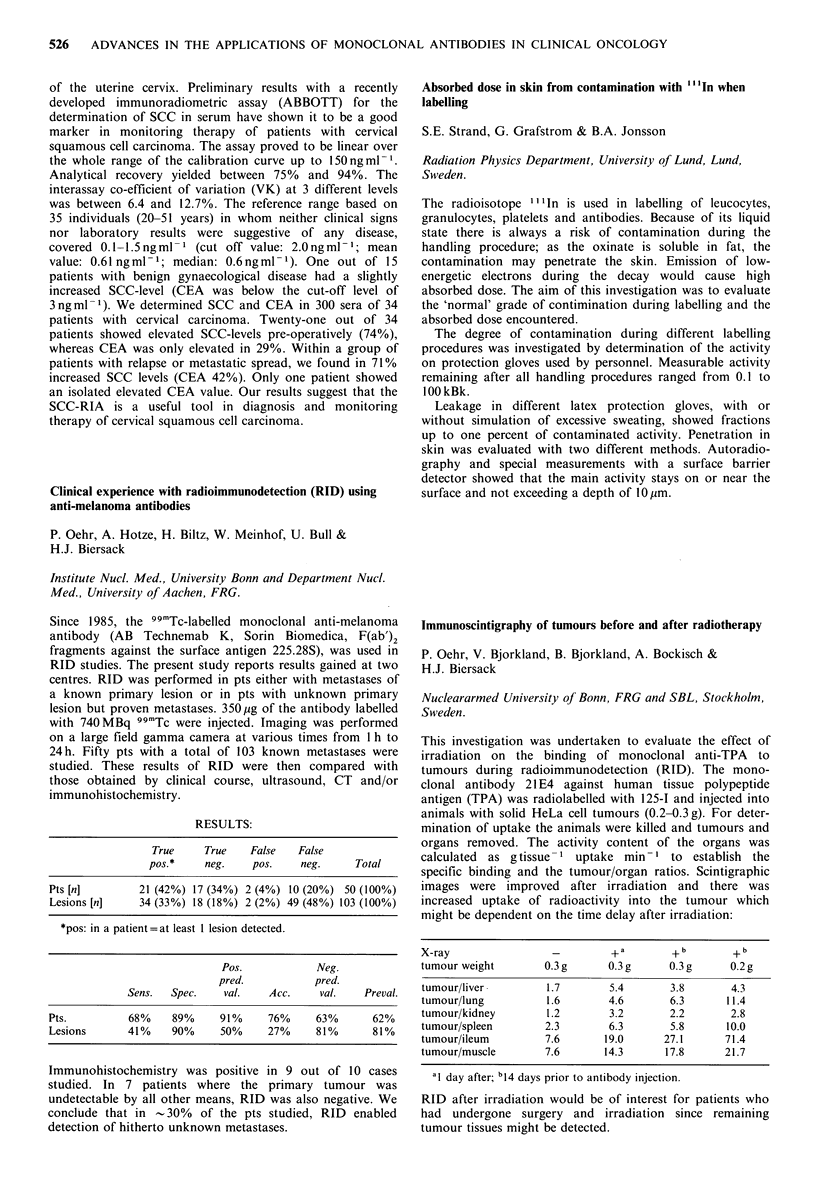

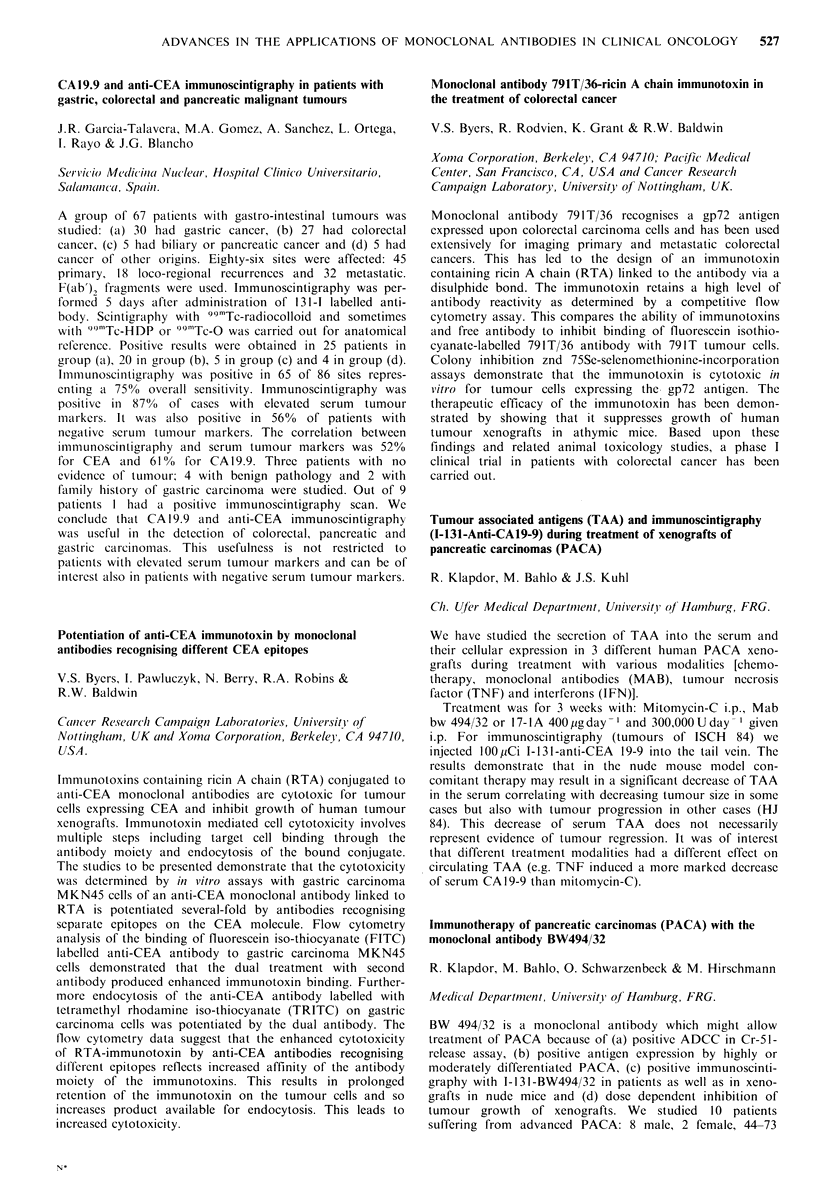

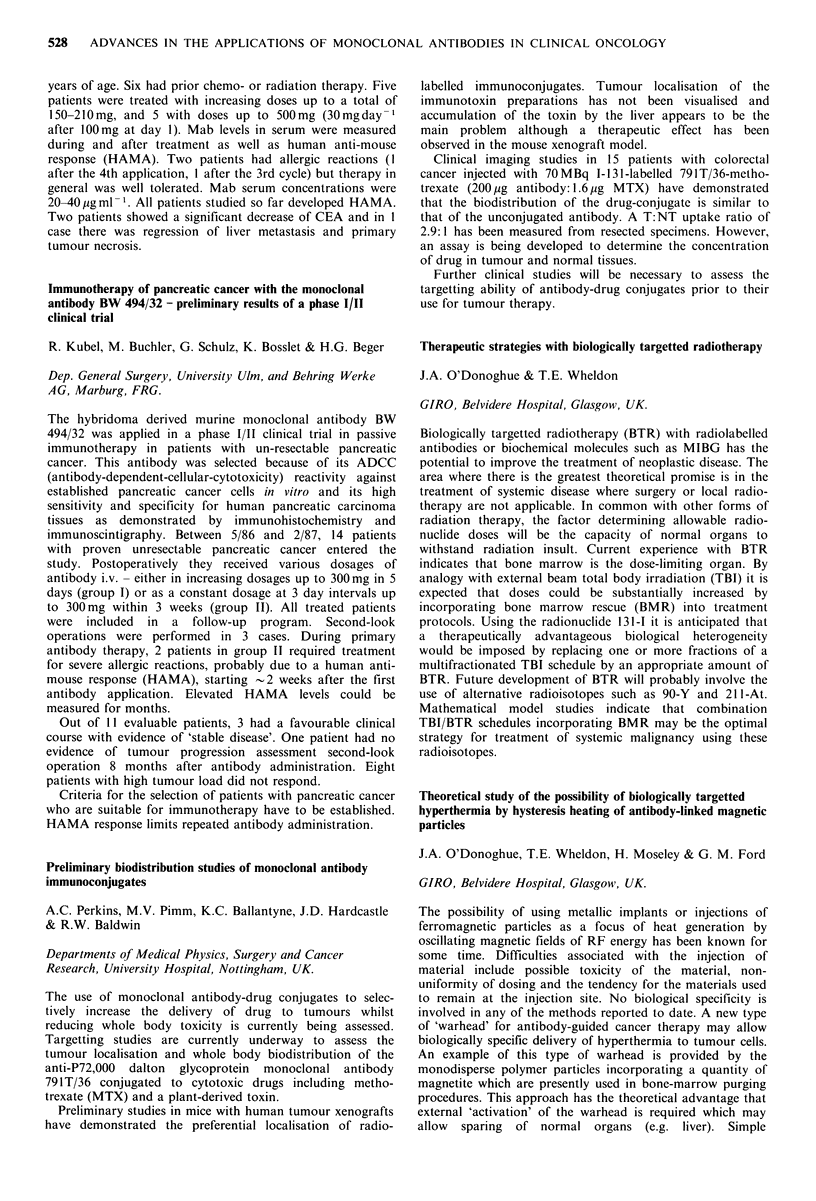

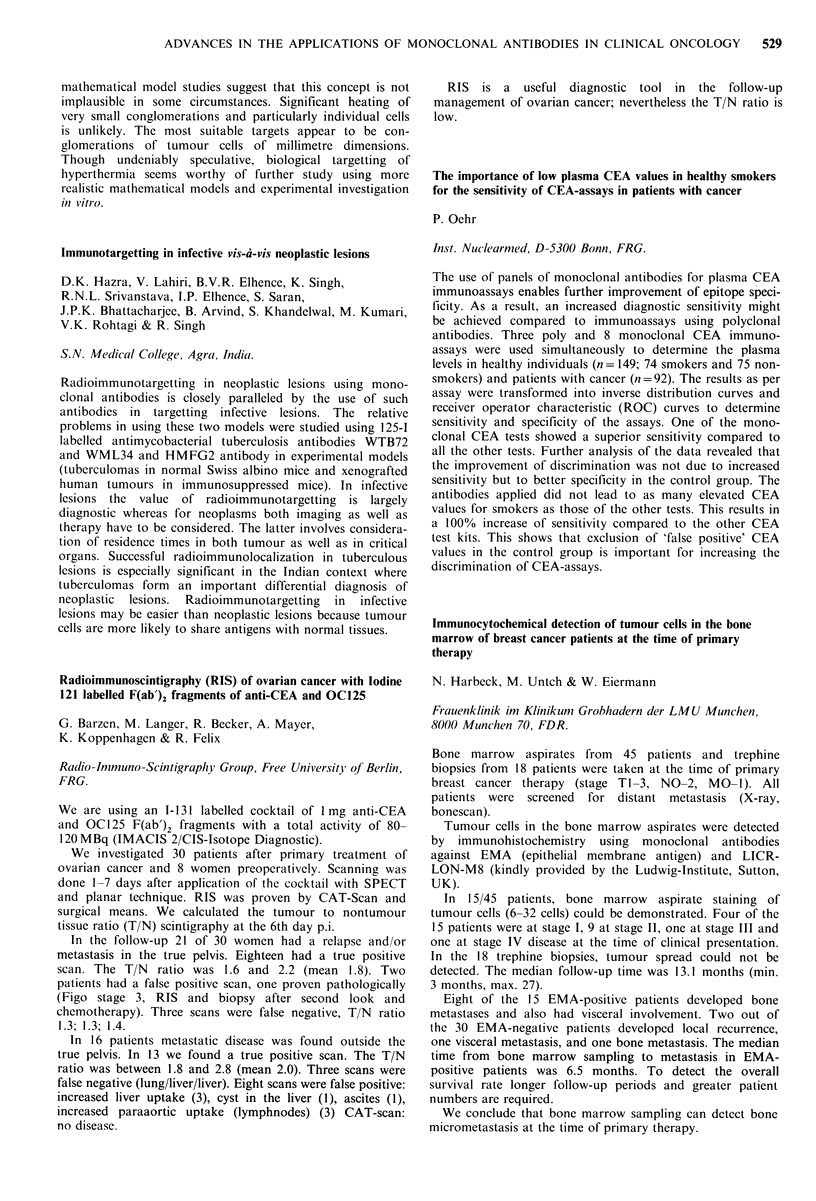

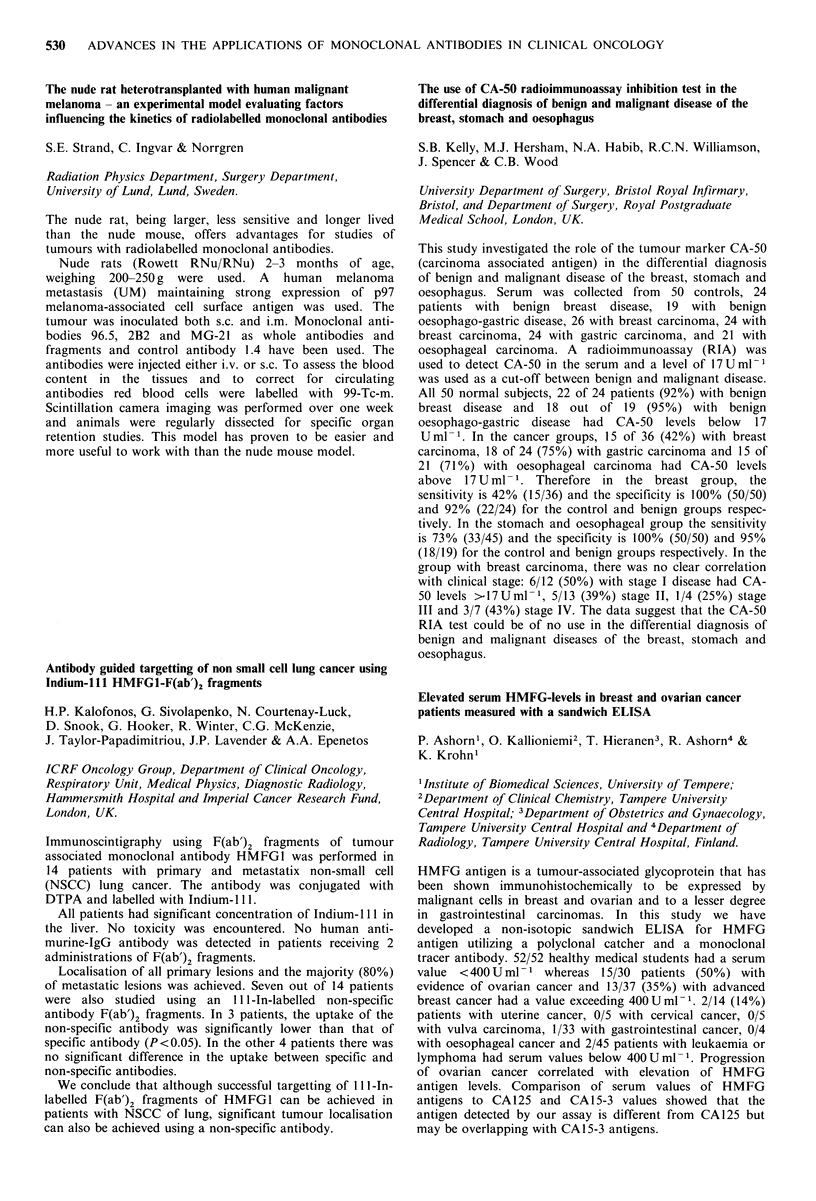


## References

[OCR_00370] Delaloye B., Bischof-Delaloye A., Buchegger F., von Fliedner V., Grob J. P., Volant J. C., Pettavel J., Mach J. P. (1986). Detection of colorectal carcinoma by emission-computerized tomography after injection of 123I-labeled Fab or F(ab')2 fragments from monoclonal anti-carcinoembryonic antigen antibodies.. J Clin Invest.

[OCR_01141] Glassy M. C., Handley H. H., Hagiwara H., Royston I. (1983). UC 729-6, a human lymphoblastoid B-cell line useful for generating antibody-secreting human-human hybridomas.. Proc Natl Acad Sci U S A.

[OCR_00093] Neuberger M. S., Williams G. T., Mitchell E. B., Jouhal S. S., Flanagan J. G., Rabbitts T. H. (1985). A hapten-specific chimaeric IgE antibody with human physiological effector function.. Nature.

